# Milk proteins and fat influence Ag migration from model dairy packaging containing silver nanoparticles

**DOI:** 10.1038/s41538-025-00684-5

**Published:** 2026-01-13

**Authors:** Laxmi Adhikari, Srushti B. Pansare, Rakesh R. Mudireddy, Monisha Srinivasan, Timothy V. Duncan

**Affiliations:** 1 Human Foods Program, US Food and Drug Administration, Bedford Park, IL 60501 USA; 2 Department of Food Science and Nutrition, Illinois Institute of Technology, Bedford Park, IL 60501 USA

**Keywords:** Biochemistry, Biotechnology, Microbiology, Nanoscience and technology

## Abstract

We investigated migration of Ag from model Ag nanoparticle (AgNP)-loaded polyethylene films into bovine milks with varying milkfat content after storage for 10 days at 20 °C. Ag migration into 2% fat milk (2.18 ± 0.03 ng/cm^2^) was comparable to that observed in skim milk (2.16 ± 0.14 ng/cm²), while whole milk (4% milkfat) had the lowest migration (1.80 ± 0.07 ng/cm²). Notably, Ag migration into skim, 2%, and whole milk was 1.72, 1.69, and 1.40 times higher, respectively, than that into 50% aqueous ethanol, a common simulant for whole milk. At least a portion of the migrated Ag in milk existed as nanoparticles, suggesting that milk components influence the final form of migrated Ag. We explored the behavior of Ag^+^ ions in milks and observed efficient Ag^+^ transformation to Ag^0^ NPs and Ag_2_O NPs. Electron microscopy images revealed polydisperse, quasi-spherical Ag particles with sizes ranging from 5 to 70 nm. Milk proteins, particularly casein and whey, play a role in the transformation of dissolved Ag^+^ to nanoparticles, while lactose influences the nanoparticle composition. These findings highlight that Ag interactions with milk components affect Ag migration dynamics and emphasize the need for a better delineation of appropriate food simulants for migration studies with AgNP-containing polymers.

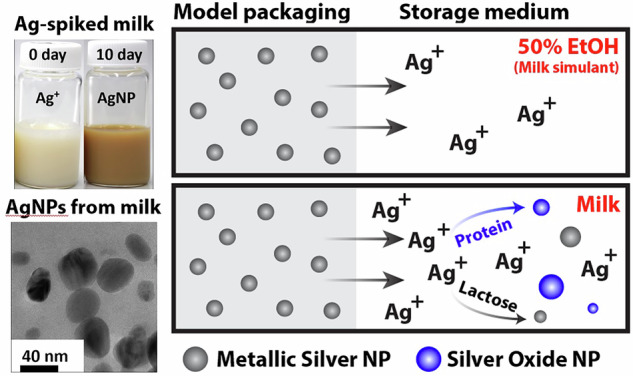

## Introduction

Food packaging containing silver nanoparticles (AgNPs), which are known for their potent, broad-spectrum antimicrobial activity against various pathogens^1–8^, may offer a promising solution to extend the shelf life of foods, reduce food waste, and mitigate the risk of foodborne illnesses, particularly in highly perishable products such as fruits/vegetables^9–11^, meats^12–15^, and dairy^6,16–19^. Yet, despite numerous studies establishing efficacy, antimicrobial AgNP-containing packaging for food products has struggled to move beyond the basic research phase, in part due to a poor understanding of its safety under specific conditions of use.

In the United States and other nations that have strong legal frameworks to ensure the safety of commercial food products, premarket authorization is required before new food packaging materials can be brought to market. Manufacturers are generally required to provide regulatory authorities with data establishing that their materials are safe under the intended conditions of use, and these data are then evaluated by qualified experts to determine whether the statutorily defined safety standard has been met. A key component of the safety assessment is an evaluation of how much of a food contact substance migrates from a packaging material to a food under intended use conditions. The Food and Drug Administration (FDA) in the United States and its counterparts in other countries have established recommended procedures and guidelines to help manufacturers perform experimental migration assessments, and these methods have repeatedly been shown to give reliable but conservative projections of migration for a broad range of organic polymer additives and contaminants. Unsurprisingly, assessments of AgNP migration from polymers published in the primary literature have relied on these testing recommendations as well^20–25^, even though these recommendations are primarily based on diffusion data for low molecular weight organic polymer additives. Our previous work has shown that mass transfer of nanoscale materials out of polymers likely operates under a dissolution-based paradigm, which is very different than the diffusion-based paradigm that determines migration of most organic molecules^26–28^. This raises the question of whether migration testing protocols developed for organic molecules generate reliable but conservative predictions of migration for AgNPs and other metal- or metal-oxide-based engineered nanomaterials. Without data to support the reliability of experimental migration measurements for AgNP-containing materials, and a more robust understanding of the environmental and chemical factors (polymer, particle, or food) that may influence AgNP migration under a variety of circumstances, it is difficult to confidently establish the safety of AgNP-containing polymers for food contact use.

Although migration tests can be done with real foods, in practice, migration studies are commonly conducted using food simulants, which are solvents that broadly represent the chemical properties of foods and are simpler to analyze. Recommended simulants depend on the intended use of the packaging material and are tabulated in the FDA’s publicly available chemistry guidance document^29^. Our work on AgNP-containing food contact polymers over the last few years has focused on determining whether certain food substances may influence AgNP migration, with the aim of identifying situations in which following the current food simulant recommendations could lead to under- or overestimation of potential consumer exposure. Simulants are reasonable models of real foods for the purposes of assessing migration of low MW organic molecules out of polymers because, in most relevant diffusion models, solubility of the organic migrant in the food (relative to that in the polymer, i.e., the partition coefficient) is one of the predominant factors that determines the concentration of migrant in the food, particularly as the system approaches equilibrium. Thus, appropriate food simulants are frequently those that have similar solubility properties to the food they are intended to represent, and they can be easily established through migration experiments using a variety of organic surrogate compounds. For AgNPs, however, we have found that Ag migration from AgNP-containing polymers is dependent on additional factors, including complex chemical interactions between migrated Ag ions and food components that may not be accounted for when using common food simulants^30,31^. For instance, food substances with reducing properties can facilitate redox chemistry with migrated Ag^+^ ions and drive conversion of migrated Ag^+^ back into metallic AgNPs within the food matrix, which maintains large Ag^+^ concentration gradients that enhance total Ag migration compared to simulants that lack these ingredients^30^. With their innate permeation into polymers, certain organic acids can influence polymer-embedded NP surface oxidation and oxide layer shedding^28^, while food additives like TiO_2_ can mediate the interaction of Ag with other food components like sucrose, increasing migration and altering the form of the Ag in the simulant^31^. Inorganic sulfides in the simulant environment, on the other hand, can suppress migration by protecting AgNP surfaces from oxidative dissolution^32^. In these situations, simple solvents do not account for Ag-food interactions that may influence migration, suggesting that selecting simulants based only on hydrophobicity/hydrophilicity considerations may be insufficient to predict migration of AgNPs from nanotechnology-adapted food contact polymers.

In the current study, we evaluated Ag migration from AgNP-containing polymers into bovine milk and compared the migration to commonly recommended milk simulants. Dairy products are rich in proteins, fats, natural and artificial sugars, and other inorganic and organic substances, many of which may have strong interactions with Ag that can influence Ag migration out of polymers containing AgNPs^33–37^. The FDA’s 2007 Chemistry Guidance recommends the use of 10% aqueous ethanol as a simulant for Type IV-B foods, which includes oil-in-water emulsions like milk. However, recent evaluations of ethanol-water mixtures and their correlations to real food-polymer partition coefficients have suggested that a higher ethanol content (up to 50-60% v/v) may be more appropriate for migration assessments in milks under certain conditions^38–40^. These findings have prompted the FDA to suggest that 50% ethanol may be a more appropriate simulant for dairy products and other similar foods, including infant formula and human milk. As with other food simulant guidance, however, these recommendations have largely been formulated based on diffusion data for small organic molecules. Given the potential sensitivity of Ag to sugars, proteins, and fats in milk, we hypothesized that currently-recommended ethanol-water mixtures may not provide reliable and conservative representations of the Ag migration dynamics into milk. To test this hypothesis, we synthesized AgNPs and incorporated them into low-density polyethylene (LDPE) cast films. Then, we investigated Ag migration from these films into bovine milk samples of varying milkfat content and ethanol-water dairy simulants. Additionally, to better understand Ag-milk chemistry that may influence migration dynamics and the reformation of AgNPs from migrated Ag^+^, we examined the AgNP-forming behavior of Ag^+^ ions in different milks and, separately, in protein and sugar solutions over a range of Ag concentrations. Specifically, we examined the roles of the major milk proteins, casein and whey, along with lactose, the primary carbohydrate and a key reducing sugar in milk.

## Results and discussion

### Migration of Ag out of AgNP/LDPE film and into bovine milks and milk simulants

Our primary objectives were to determine the migration of Ag from AgNP-incorporated low-density polyethylene (AgNP/LDPE) packaging into bovine milks, to evaluate the reliability of solvents (milk simulants) commonly used to assess migration from AgNP-containing polymers into dairy products under refrigerated storage conditions, and to identify the underlying factors that influence Ag migration into milks from AgNP-containing polymers. To achieve this, we synthesized oleylamine (OA)-capped AgNPs, followed by a ligand exchange using polyethylene glycol thiol (PEG-SH-2000), and incorporated the modified AgNPs into LDPE cast films through melt-compounding to fabricate AgNP/LDPE films for migration testing. The AgNPs were synthesized following a previously established method^41^. Ag concentration in the AgNP/LDPE film was determined to be 0.050 ± 0.001 mg per g of polymer.

We followed a previously published protocol^42^ to assess the migration of Ag from AgNP/LDPE films into milks under refrigerated storage conditions. Analogous experiments assessing migration into selected ethanol-water milk simulants were also performed. Briefly, AgNP/LDPE was cut into circles, and the circles were then stored fully submerged in milks and simulants at 20 °C for 10 days, after which the milks and simulants were assayed for Ag content by ICP-MS in standard mode. The time-temperature conditions are intended to represent long-term storage under refrigeration. Although refrigeration temperature is generally understood to be 4 °C (40 °F), performing migration tests at 4 °C for weeks or months would be burdensome. As a result, for migration experiments, accelerated testing at 20 °C for 10 days has been recognized to be roughly equivalent to longer term storage at 4 °C^29^. We chose to study migration under accelerated long-term refrigeration conditions because, in our experience, UHT grade milk may be stored in the refrigerator for several weeks after opening without appreciable degradation of its quality, so this represents a potential worst-case scenario for milk storage. In our accelerated tests at 20 °C for 10 days, we observed no signs of spoilage (off-odors, coagulation) and only a small change in pH for milks stored in either the presence or absence of Ag cations (Supporting Information, Table S1). Three types of milk with different fat content were examined in these experiments, including skim milk (< 0.5% fat), 2% fat milk, and whole milk (nominally 4% fat). The simulants were water, 10% aqueous ethanol, and 50% aqueous ethanol.

**Fig. 1:**
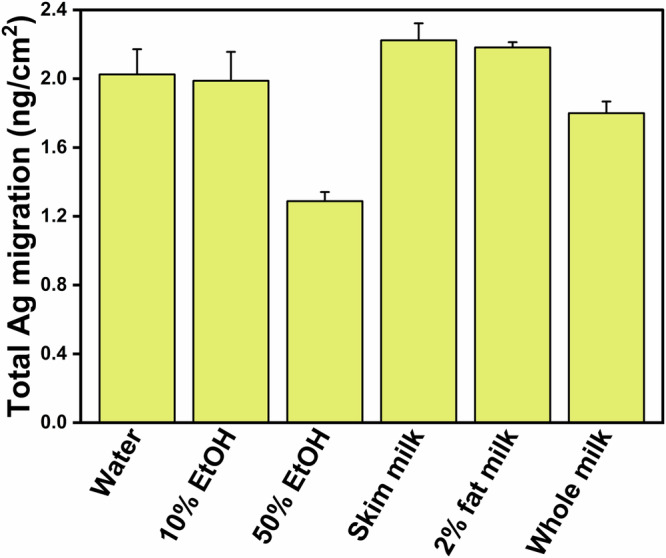
Ag migrated from AgNP/LDPE film sections into bovine milks and bovine milk simulants (water, 10% aqueous ethanol, 50% aqueous ethanol) after 10 days of storage at 20 °C. The films were sectioned into 42 mm diameter circles, and 25 mL of each simulant was used for the migration tests. All data points represent the averages of four independent samples, with error bars indicating standard deviations.

Figure 1 plots total Ag mass migrated out of the AgNP/LDPE film sections into different milks and milk simulants after 10 days storage at 20 °C. A notable feature of the migration results is the inverse correlation between Ag migration and the hydrophobicity of the contacted environment. Migration into the ethanolic-water simulants dropped markedly as the % ethanol increased, as did the migration into milks as the milkfat content increased. Among the three simulants and the milks examined, Ag migration was lowest in 50% aqueous ethanol (1.29 ± 0.05 ng/cm^2^ of packaging surface area) and highest in skim milk (2.22 ± 0.10 ng/cm^2^). Migration into 2% fat milk (2.18 ± 0.03 ng/cm²) was indistinguishable from that observed in skim milk, while whole milk exhibited significantly lower migration (1.80 ± 0.07 ng/cm², p < 0.05). Migration into water and 10% aqueous ethanol, 2.02 ± 0.15 and 1.99 ± 0.17 ng/cm^2^, respectively, was higher than into 50% aqueous ethanol solution, but lower than in skim and 2% fat milks. Notably, the Ag migration into skim milk, 2% milk, and whole milk was 1.72-fold, 1.69-fold, and 1.40-fold greater than the migration into the 50% aqueous ethanol simulant (Table S2). This suggests that using a 50% ethanol-water solution as a milk simulant may underestimate Ag migration into bovine milks, particularly when the milkfat content is low. Simulants containing lower ethanol content may provide a closer estimate of total Ag migration for AgNP/LDPE into milks, although Ag migration was still underestimated in some cases. Note that the levels of migrating Ag that we measured here align with other studies on AgNP migration from polymers, which generally report Ag migration at low concentrations (sub-ppb levels)^43,44^. For example, studies of commercial Ag-enabled packaging (Ag content 0.01–0.023 mg/g) measured migration into 10% ethanol at 4–5 ng/cm², consistent with our findings^45^.

To assess the transformation of Ag^+^ ions into Ag nanostructures in the migration samples, we conducted separate analyses of milk and milk simulants using SP-ICP-MS. We note at the outset that SP-ICP-MS analysis of undigested milk proved challenging due to adherence of fats and/or proteins to the ICP-MS tubing. Because of this phenomenon, the SP-ICP-MS data could not be used to reliably quantify concentrations or size statistics of Ag nanostructures formed in milks after migration. This limitation should be kept in mind when trying to make quantitative comparisons of the SP-ICP-MS data obtained in milks, although qualitative comparisons of particle concentrations are certainly reasonable when the differences in calculated concentrations between samples are very large. With this limitation in mind, the SP-ICP-MS time scans of ^107^Ag for migration samples, as shown in Fig. S3, revealed the presence of a small number of Ag particles in water and in aqueous ethanol solutions, while much higher particle counts were observed in all milk types. This suggests the transformation of a non-negligible fraction of migrated Ag^+^ ions into Ag nanostructures in milks. Among the milks, Ag particle counts appeared to be more prevalent in skim milk than in milks containing more than trace levels of fat (2% and whole), implying that fats might interfere with or inhibit Ag particle formation. Regardless of the difficulty of quantifying AgNP concentrations in the different milk samples due to the limitations noted above, the SP-ICP-MS time scans nevertheless strongly indicate that reformation of Ag nanostructures in milks stored in the presence of AgNP/LDPE packaging is ubiquitous and significant; this particle formation from migrated Ag^+^ is not observed in ethanolic simulants, which may explain why ethanolic simulants appear to underestimate Ag migration into milk from AgNP/LDPE. Therefore, we conclude that ethanolic simulants may both underestimate Ag migration into milk and fail to replicate the migrated Ag form.

Before discussing Ag-milk interactions, it is important to note some of the challenges that we encountered when determining accurate Ag migration data into milk, as this provides some necessary context when comparing our results to earlier work. Some additional details of the method development process are provided in the Methods section. In brief, early migration tests revealed inconsistent results that were due to a portion of the migrated Ag, along with milk fat and protein, adhering to both the surface of the film and the migration cell walls (50 mL polypropylene tubes). This portion of the migrated Ag was generally not accounted for when simply decanting milk from the storage tubes and rinsing the tubes/films with water. More accurate and reproducible measurement of migrated Ag was achieved by rinsing the test films and the migration cells with ethanol and concentrated HNO₃, respectively. The residues from the evaporated ethanol rinse were then added back into the milk prior to digestion and analysis. Additionally, because milk contains trace levels of chloride, it was necessarily to prevent soluble Ag losses due to AgCl precipitation in milk^46^. This was accomplished by adding HCl to the digestates to promote the formation of soluble AgCl_*x*_^(*x*-1)−^ complexes and prevent AgCl precipitation^47–50^. We note that prior studies of Ag in milk may not have taken steps to account for Ag loss due to AgCl precipitation. In such cases, determined Ag concentrations are likely to be erroneously low. Importantly, some previous studies of Ag migration into milks or ethanolic simulants from Ag-containing polymers have reported very low to negligible Ag migration, in contrast to our results here. If these studies did not account for AgCl precipitation or Ag loss to adsorption on plastic surfaces, then this may explain why they did not observe Ag migration, and their results may need re-evaluation. Our method development steps here demonstrate that great care needs to be taken to accurately assess Ag migration from Ag-containing packaging into milks or other foods containing levels of chloride likely to promote AgCl precipitation. Since fat and protein adherence to the plastic surface likely increases as the milk ages and emulsion stability decreases, these steps may become especially important when assessing migration after longer storage times.

### Fate of Ag⁺ ions in different milk samples

**Fig. 2:**
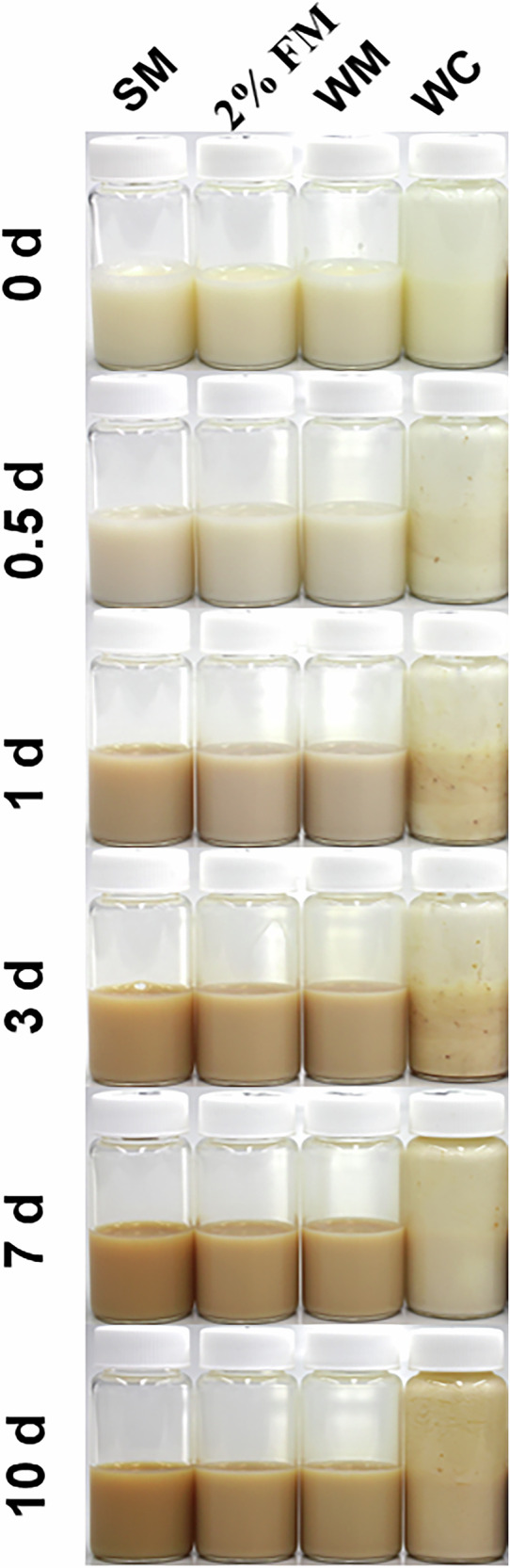
Photographs of bovine milk spiked with AgNO₃ and stored at 20 °C under ambient light for different time intervals. Each milk type (SM: skim milk, 2% FM: 2% fat milk, WM: whole milk, WC: whipping cream) was spiked with 100 µL of AgNO₃ stock solution (12.5 mg/mL) and gently vortexed for 5 s. The vials were then placed in a Thermo Max Q4000 orbital shaker set at 60 rpm and 20 °C. The evolving colors represent the gradual formation of milk-formed AgNPs (m-AgNPs) starting as early as 12 h after the Ag^+^ addition. The brown hue intensified until the 7^th^ day of incubation for most of the milk types, indicating the progress of m-AgNP formation.

Previous studies have shown that proteins, lactose, and vitamins, all commonly found in milk, are capable of transforming Ag⁺ ions to nanoparticulate Ag under different conditions^51–54^. In fact, some studies have used bovine milk as eco-friendly media for the biosynthesis of AgNPs^55–57^. While these studies have optimized experimental conditions to produce high-quality AgNPs (elevated temperatures and pH values), we sought to evaluate whether Ag^+^ ions interact with milk components under the same conditions we used to study Ag migration, and what the fate of those interactions is. We started our investigation by adding AgNO₃ to various milks to achieve a final Ag concentration of 0.73 mM and incubated the mixtures at 20 °C while monitoring the solutions for color changes signifying AgNP formation. This Ag concentration is higher than typically observed in migration experiments but facilitated visual observation of Ag nanostructure formation we hypothesized may result from Ag⁺-milk interactions. Figure 2 displays a series of photographs of Ag-spiked milk samples after storage at 20 °C, over varying time intervals following Ag⁺ addition. In addition to the milks that we studied in the migration experiments, we have also included a heavy whipping cream (> 30% milkfat) to determine with Ag–milk interactions are still manifested at very high fat content. All milk samples were initially white and slowly transitioned to a brown color within a few hours. This color change, which became evident as early as 12 h post Ag⁺ addition, and intensified until the 7^th^ day, suggested a strong interaction of Ag^+^ ions with milk resulting in the gradual formation of AgNPs^58^. We will designate AgNPs formed spontaneously when Ag^+^ is introduced into milk or into aqueous dispersions of milk components as m-AgNPs to distinguish them from purely metallic AgNPs that we deliberately synthesized using laboratory reagents (chemical reductants and capping agents) and then incorporated within LDPE. Notably, color changes associated with m-AgNPs were not observed in Ag⁺-spiked milk after storage under dark conditions (Fig. S4), indicating that spontaneous transformation of Ag⁺ ions to m-AgNPs in milk is photoactivated.

**Fig. 3:**
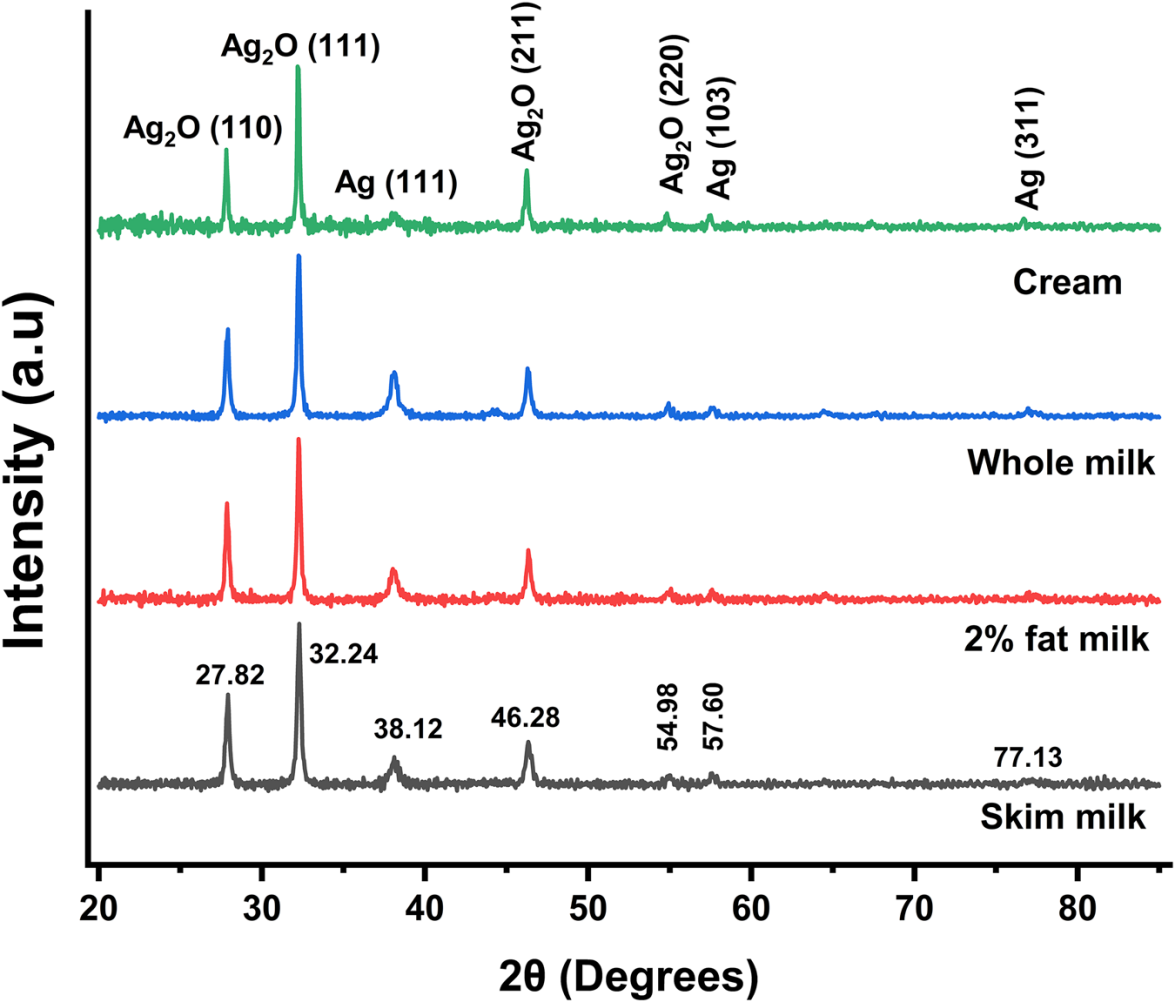
X-ray diffractograms of nanoparticulate Ag extracted from milks 10 days after spiking with AgNO_3_. A mix of Ag_2_O and Ag^0^ NPs were observed in all milk samples. The storage temperature was 20 °C.

**Fig. 4:**
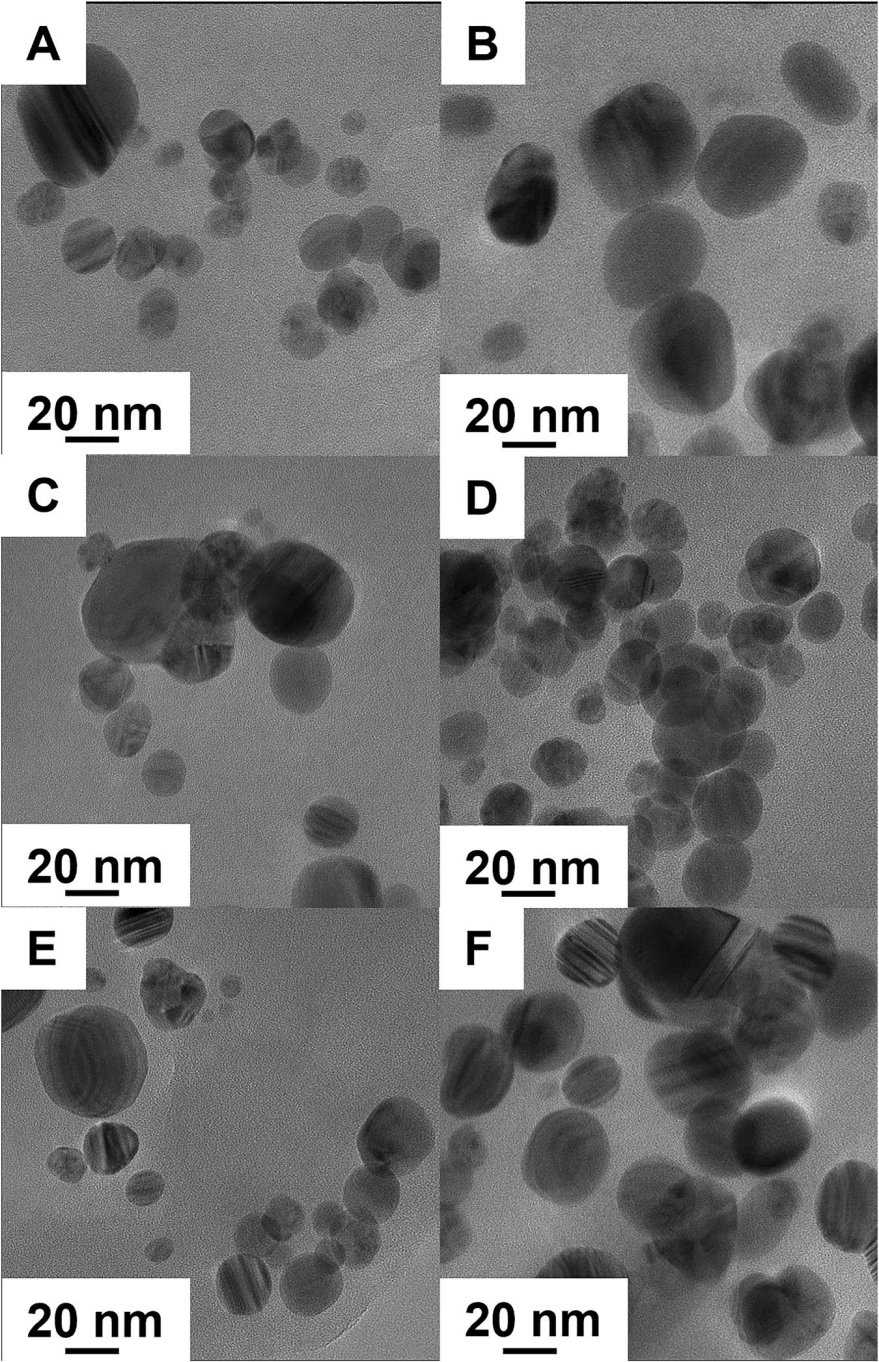
TEM images of m-AgNPs extracted from different milks after 10 days of incubation with AgNO_3_ at 20 °C. Panels (**A**) and (**B**) correspond to skim milk, panels (**C**) and (**D**) correspond to 2% fat film, and panels (**E**) and (**F**) correspond to whole milk. After incubation, m-AgNPs were separated from the milk matrix by centrifugation and resuspended in deionized water. m-AgNPs extracted from whipping cream had large amounts of organic matter adsorbed onto the AgNP surfaces, impeding TEM imaging.

The m-AgNP formation was confirmed by UV–Visible spectroscopic analysis. On the third day of incubation, UV–Visible absorption spectra of the stored samples were collected using the relevant pure milk in the reference channel. Broad absorption bands in the 350-600 nm range (Fig. S5) were observed for each sample, consistent with a surface-plasmon resonance (SPR) feature typical of Ag nanostructures^59^. Bathochromic shifting of the SPR peak maxima as a function of increasing milkfat % suggests that fats influence the m-AgNP growth kinetics, with higher fat content resulting in larger particles^60^. X-ray diffractograms of m-AgNPs isolated from milks (Fig. 3**)** showed characteristic 2-theta peaks at 27.82°, 32.24°, 46.28°, and 54.98°, corresponding to the (110), (111), (211), and (220) lattice planes of Ag₂O NPs^61^. Additionally, less prominent peaks at 38.12°, 57.60°, and 77.13°, associated with the (111), (103), and (311) lattice planes of metallic Ag, were also observed^41^. XRD analysis therefore showed that m-AgNPs are composed of mixtures of Ag^0^ and Ag_2_O NPs after incubation at 20 °C for 10 days, with Ag_2_O NPs predominating in all cases. No clear trends were observed in m-AgNP composition as a function of milkfat content. Interestingly, purging the milk with a gentle flow of nitrogen before spiking with AgNO_3_ resulted in enhancement of metallic Ag^0^ NP peaks (Fig. S6), suggesting m-AgNP composition may be influenced by environmental conditions in the package headspace. Ag^+^ reduction to metallic m-AgNPs appears to be more likely in the absence of oxygen. Mean NP diameters estimated using the Scherrer equation^62^, based on the corrected half-width of the (111) peak for Ag_2_O, yielded values ranging from 25 to 29 nm across all types of milk, while TEM images showed that the m-AgNPs were polydisperse, with diameters ranging from 9 to 45 nm (Fig. 4).

### Interactions between Ag⁺ ions and lactose and/or milk proteins

**Fig. 5:**
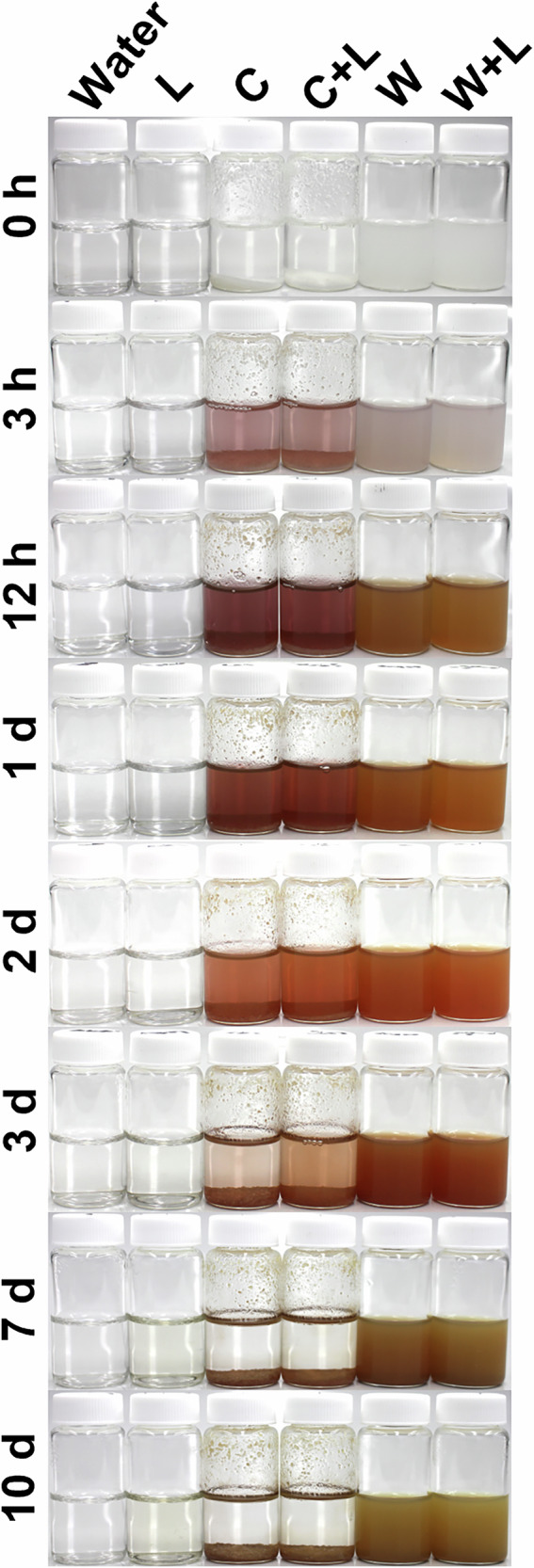
Photographs of lactose and protein (casein and whey) dispersions spiked with AgNO₃ and stored at 20 °C under ambient light for different time intervals. L refers to lactose, C refers to casein, and W refers to whey. Each dispersion was spiked with 100 µL of AgNO₃ stock solution (12.5 mg/mL), briefly vortexed for 5 s, and placed in an orbital shaker (60 RPM, 20 °C). The observed color changes indicate the gradual formation of milk component-formed AgNPs (m-AgNPs), beginning as early as 3 h after the addition of Ag⁺. In the casein dispersion, m-AgNPs began settling by the 2^nd^ day, whereas the m-AgNPs in whey remained suspended throughout the incubation period, likely due to the higher solubility of whey in water compared to casein. Lactose showed the least m-AgNP formation, noticeable only on the 7^th^ day of incubation.

We demonstrated that Ag⁺ ions undergo dramatic changes in bovine milks after incubation at 20 °C for 10 days, leading to efficient formation of m-AgNPs. To determine which milk components may drive this chemistry, and whether reducing sugars in milk play a role, we dispersed 295 mg of commercial casein protein powder or 63 mg of commercial whey protein powder in 10 mL of DI water. Similarly, we dissolved 500 mg of lactose powder either in these protein dispersions or in neat water. The whey, casein, and lactose concentrations were selected to replicate their approximate concentrations in bovine milk^63^. (Throughout this section and the next section, “whey dispersions” and “casein dispersions”, or similar expressions, should be understood to refer to aqueous dispersions of commercial whey or casein protein powders at the above-specified concentrations, respectively.) Finally, we spiked casein, whey, lactose, and their respective mixtures with AgNO₃ (final Ag concentration of 0.73 mM). We then incubated the mixtures at 20 °C and monitored the solutions for color changes indicative of m-AgNP formation. Color changes in the protein dispersions, both with and without lactose, were observed as early as 3 h of incubation, indicating the onset of m-AgNP formation (Fig. 5). In the casein dispersion, the characteristic wine-red color of m-AgNPs intensified over the following 24 h, with sedimentation occurring by the second day. In contrast, the orange hue of m-AgNPs in the whey dispersion continued to intensify until the third day, while the particles remained suspended throughout the incubation period, likely due to the higher solubility of whey in water compared to casein. Differences in m-AgNP color in casein and whey dispersions likely reflect differences in m-AgNP particle size distributions formed in each case. Lactose exhibited the least m-AgNP formation, with color associated with m-AgNP formation only (barely) observable by the seventh day of incubation. After 7 days, the vibrant colors of m-AgNPs in both whey and casein dispersions transitioned to a brown color and m-AgNPs largely sedimented out of solution, suggesting pronounced aggregation. As in the case of Ag^+^ added into milk (Fig. S4), color changes associated with m-AgNP formation were not observed when Ag^+^-spiked protein solutions were stored in the dark overnight, again implying that m-AgNP formation in the presence of proteins is photoinduced. Light/dark conditions did not affect m-AgNP formation in the presence of lactose.

Formation of m-AgNPs was corroborated by UV–Visible spectroscopic analysis. After 24 h of incubation, UV–Visible spectra of the samples were recorded, using water as the reference. The presence of broad absorption bands in the 350–700 nm range (Fig. S7), similar to those observed in bovine milk, is indicative of SPR features signifying m-AgNP formation. Notably, spectral shapes and peak maxima of m-AgNPs formed in the casein-containing dispersions differ from those formed in the whey-containing dispersions, consistent with color differences noted above. XRD (Fig. S8), revealed that m-AgNPs formed in aqueous dispersions of both whey and casein powder were predominantly composed of Ag₂O NPs, whereas m-AgNPs formed in pure lactose solution were primarily metallic Ag^0^ in nature. Interestingly, m-AgNPs formed in protein dispersions containing lactose show mixtures of both oxide and metallic forms of Ag, particularly in the case of dispersions containing casein. We note that aqueous dispersions containing casein but no lactose also tended to form some metallic Ag^0^ NPs at longer storage times as well (24 h vs 4 h). As lactose alone does not appear to be effective at driving m-AgNP formation, we interpret these XRD results to mean that lactose influences the activity of protein-driven m-AgNP synthesis in the protein dispersions when it is present rather than acting independently. The UV-Vis results (Fig. S7) showed that the presence of lactose in the protein-lactose mixtures had minimal effect on the solution appearance compared to analogous solutions without lactose, except for some slight evidence of m-AgNP stabilization (e.g., C + L D3 sample), which might be taken to imply that lactose plays very little role in m-AgNP formation in milks. The XRD results also suggest that lactose has minimal influence in driving m-AgNP formation, but it evidently plays a role in modulating the composition of the formed m-AgNPs, most likely through its activity as a reducing agent.

### The role of milk proteins in Ag transformations

The rapid conversion of Ag^+^ ions to m-AgNPs in aqueous protein dispersions suggests that proteins, among all milk components, play a key role in the transformation of Ag^+^ to m-AgNPs. Proteins contain functional groups such as disulfide bonds, free thiols, and branched-chain amino acids with negatively charged groups^64^, which interact strongly with Ag^+^ ions. Rodzik et al. observed that Ag^+^ ions interact with β-lactoglobulin (βLG), a globular protein found primarily in the whey fraction of bovine milk, via a three-step binding process: rapid initial sorption on the external surface, slower internal sorption into the protein structure, and eventual equilibrium^35^. At Ag concentrations higher than 150 mg/L, Rodzik et al. observed that the interaction led to the formation of Ag nanostructures. This process involved the binding of Ag⁺ ions with the carboxylate, amino, phenolic, and thioether groups of the amino acid residues, which induced structural changes in the protein. These interactions facilitated the formation of Ag nanoclusters within the protein network, creating unstable nucleation sites that subsequently coalesced and grew into larger AgNPs through Ag diffusion. Several other studies have explored the interaction of Ag^+^ ions and proteins such as α-lactalbumin^37^, casein^36^, and lactoferrin^65^, observing similarly strong Ag-protein interactions. The high distribution coefficient (*K*_d_) exhibited by these proteins for Ag sorption further suggests large binding affinities between Ag and the proteins.

**Fig. 6:**
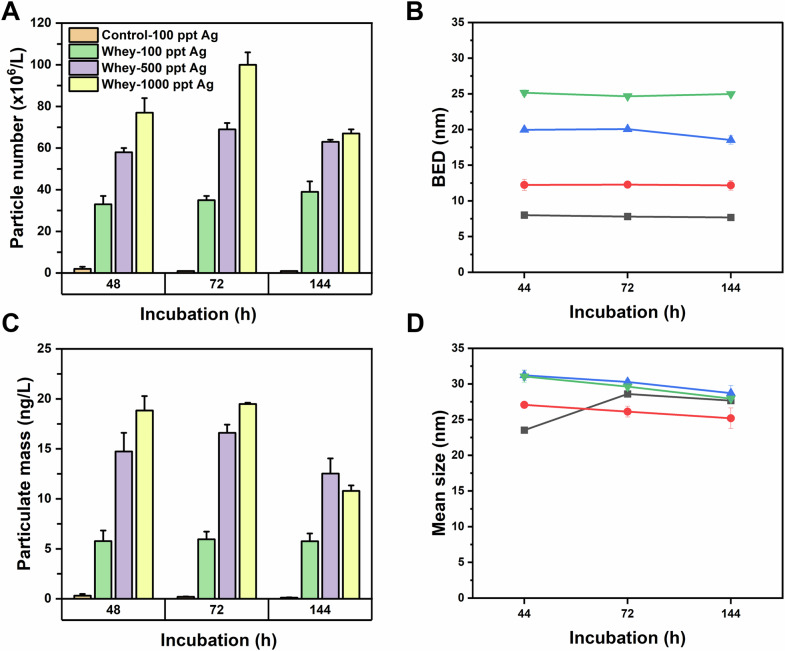
SP-ICP-MS analysis of AgNPs formed in whey dispersions (6.3 g/L) spiked with Ag^+^ ions. The top panels (**A**, **B**) show particle number concentrations and mass concentrations, respectively. The bottom panels (**C**, **D**) display the background equivalent diameters (BEDs) and mean particle diameters. Each data point represents the average of three independent samples, with error bars indicating standard deviations. The legend in panel (**A**) also applies to panel (**C**). In panels (**B**) and (**D**), the black line represents the water control containing 100 µg/L Ag⁺ ions while red, blue, and green lines correspond to whey dispersions spiked with 100, 500, and 1000 µg/L Ag⁺ ions, respectively.

Our experiments on the behavior of Ag in aqueous dispersions of commercial whey protein powder, discussed below, were consistent with many findings from past studies. ICP-MS analysis of unbound Ag^+^ fractions in aqueous mixtures of AgNO_3_ and dispersed whey protein indicated rapid adsorption of Ag into dispersed whey protein complexes, reaching equilibrium within a few minutes of mixing (Fig. S9 and Table S3). Similarly, we observed efficient m-AgNP formation in aqueous whey dispersions within 48 h of Ag^+^ spiking (Fig. 6), which we tracked by SP-ICP-MS. Aqueous dispersions of whey protein powder (6.3 g/L) with trace Ag^+^ concentrations ranging from 100 µg/L to 1000 µg/L were incubated at 20 °C, and samples were withdrawn at specified intervals for analysis. While the negative control with 100 ng/L Ag^+^ in water showed the occurrence of a few AgNPs, large numbers of m-AgNPs were detected as early as 48 h of incubation in the aqueous dispersions of whey protein powder. Figure 6 and Table S4 reveal that both NP count and particulate mass continued to increase until 72 h of incubation, after which they began to decrease across all three Ag concentrations. Note that the measured m-AgNP particle number and particle mass concentrations plotted in Fig. 6A, C are lower than the true values because invariably a fraction of m-AgNPs are obscured by the ionic background. The SP-ICP-MS data show that the fraction of m-AgNPs obscured by the ionic background increases substantially as the ionic Ag spike concentration increases (see background equivalent diameters, BEDs, Fig. 6B). This implies that the disparity between true and measured AgNP concentrations gets larger as the Ag^+^ spike concentration increases, meaning that the differences in concentrations of m-AgNPs formed by whey protein among the different Ag spike concentration groups is likely much larger than is apparent in Fig. 6A, C. Thus, while care must be taken when making quantitative conclusions about how particle number and mass concentrations change from sample to sample, the trends observed in Fig. 6 are qualitatively true. Moreover, the SP-ICP-MS data unequivocally show that the transformation of Ag^+^ to m-AgNPs is highly efficient in protein dispersions, even at concentrations relevant to their migration from Ag-containing packaging.

SP-ICP-MS analysis confirmed the conversion of Ag⁺ ions to m-AgNPs when Ag⁺ is added to the aqueous dispersion of commercial whey powder. To further elucidate the influence of Ag⁺ ions on the structural organization of the whey proteins, DLS and Raman spectroscopy were employed. The size of protein assemblies when dispersed in water can vary considerably from a few nanometers to several micrometers depending on factors such as protein concentration, dispersion medium properties (pH, temperature, dielectric constant, and solute composition), and dispersion method^66–69^. In this study, commercial whey and casein protein powders were dispersed in water via gentle vortex mixing. DLS measurements showed that, under these conditions, a small fraction of whey proteins formed nanoscale assemblies with mean hydrodynamic diameter on the order of 50 nm, while the majority existed as larger aggregates with hydrodynamic size around 250 nm (Fig. S10 and Table S5). Individual molecules of native bovine beta-lactoglobulin, the most abundant protein in whey, have an estimated hydrodynamic radius on the order of 3 nm^70^, although the protein is known to self-associate into oligomers of various sizes in different media, including milk^71^. From our DLS data we can thus infer that protein aggregates in the dispersions of commercial whey powder contained at least dozens of spontaneously self-associated whey protein molecules, with the largest aggregates comprising even more. The aqueous whey protein dispersions remained relatively stable for several hours at 20 °C (Fig. S10A) before gradually coagulating into a gel-like matrix. Importantly, introducing Ag⁺ ions into the aqueous whey dispersions induced changes to how the whey proteins were self-assembled in water. The relative intensity of the scattering peak associated with the sub-100-nm protein aggregates intensified and shifted to a larger size over the course of several days, while that associated with the larger aggregates diminished (Fig. S10B). By the end of the experiment, light scattering from the Ag-spiked aqueous whey dispersions was dominated by a single scattering peak that signified a Z-average hydrodynamic diameter of 36 nm after 48 h (Table S5). This shift suggests that Ag⁺ ions induced a reorganization of the whey protein molecules in the dispersion by a kinetically controlled process. A similar trend was observed in Ag-whey dispersions prepared in pH 7.0 phosphate buffer (Fig. S11), which more closely approximates the pH of fresh milk (pH ~ 6.5–6.7) than deionized water (pH ~ 5.6). The protein aggregate size redistribution in this case, however, proceeded much faster, with a single scattering peak (Z-average hydrodynamic diameter 96 nm) appearing within 7 h of Ag⁺ addition (Fig. S11B and Table S6). We note that the spontaneous changes to dispersed whey protein structures in the presence of Ag^+^, as measured by DLS, were accompanied by a gradual progression of the whey protein dispersions from colorless to yellow, signaling the appearance of the characteristic m-AgNP plasmon band. These DLS experiments therefore show that kinetically controlled changes to the supermolecular whey protein structures in water in the presence of Ag^+^ is correlated to m-AgNP formation and growth, strongly suggesting that the two processes are associated with each other.

Analysis of the m-AgNPs formed in aqueous dispersions of whey protein incubated with AgNO_3_ was subsequently analyzed using Raman spectroscopy to probe specific whey protein-Ag interactions that may explain the DLS results. Changes to the whey protein vibrations in the presence of Ag^+^ provide evidence of direct interactions between Ag and whey protein residues, revealing changes to whey protein structure that may be associated with the spontaneous shifting of whey protein aggregate size in the presence of Ag (Fig. S12). In general, the SERS effect enhances the intensity of the amide I and amide II peaks when proteins bind to AgNPs^72^. However, if the binding induces structural disruptions of the protein, certain Raman bands could also diminish, particularly if the secondary structure is substantially altered^35,73^. In our case, the formation of m-AgNPs caused notable changes in whey protein structure, primarily a reduction in signal intensity within the amide vibration regions. Specifically, the signal at 1675 cm⁻¹, predominantly attributed to C = O stretching vibrations in the amide I region, and the signals around 1450 cm^−1^ representing a combination of N-H bending and C-N stretching vibrations, experienced a decrease in intensity in the presence of Ag^+^. Similarly, the amide III bands at 1240 cm⁻¹ and 1311 cm⁻¹, related to C-N and N-H bending vibrations as well as the C–O stretching band at 1000 cm⁻¹, also showed intensity reductions. Conversely, peaks associated with C-C skeletal stretching in the peptide backbone around 1050–1100 cm⁻¹ were intensified, demonstrating the SERS effect of m-AgNPs. These findings suggest strong Ag-protein interactions, in agreement with the Ag-protein binding studies and DLS studies described above.

Prior analyses have frequently assumed that AgNPs formed via Ag^+^ interactions with milk proteins were composed of metallic Ag without providing compositional data to support these conclusions^35,36,57^. On the contrary, m-AgNPs in our work were predominantly composed of Ag_2_O. On the other hand, m-AgNP composition was dependent on the presence of lactose. Other authors have similarly reported that the compositions of AgNPs synthesized in bio-derived media are extremely sensitive to the conditions^74^. We also observed that light was required to catalyze m-AgNP formation in both milk and in isolated protein dispersions, suggesting that proteins (possibly aromatic residues) may act as photoinitators for redox chemistry on protein-bound Ag. We therefore conclude that the composition of Ag migrated from polymers may be quite sensitive to milk composition and storage conditions.

Most of this work to understand how potential interactions between migrated Ag^+^ and proteins in milk may influence m-AgNP formation and its relation to migration phenomena in the context of food packaging was done with commercial whey protein powder. We focused on whey protein powder because we found the whey powder easier to work with (e.g., disperse in water) than relatively hydrophobic casein powder. However, Ag-spiked aqueous dispersions of casein powder also promoted the formation of m-AgNPs. We thus infer that the binding interactions between Ag^+^ and whey protein residues and the disruption of supermolecular whey protein structures that we observed in aqueous dispersions of whey protein powder probably also occur, in some form, in aqueous dispersions of casein powder. That said, it is important to recognize that the structure and behavior of whey and casein proteins in milk are probably quite different from those in aqueous dispersions of purified protein powders. For example, casein proteins form complex micellar structures in milk, and these structures are sensitive to ion (calcium) content, pH, natural protein composition, milk age, and processing history (defatting, homogenization, etc.)^75,76^. Such complexity would be expected to lead to differences in Ag ion mobility and reactivity within the various casein networks present naturally in different milks, and these differences in Ag mobility/reactivity may be particularly pronounced when considering casein micellar structure in milk as compared to casein structures formed by dispersion of purified casein powder in distilled water. We expect similar arguments can be made with respect to whey protein complexes in milk vis-à-vis aqueous dispersions of whey protein powder. Nevertheless, while Ag-protein interaction experiments reported above are certainly not intended to be a precise simulation of Ag behavior in a complex, protein-containing mixture like milk, they clearly demonstrate that Ag readily interacts with the various proteins found in milk, these interactions can rapidly induce the formation of Ag nanostructures under certain conditions, and the composition of these nanostructures can be influenced by reducing carbohydrates that may also be present.

## Discussion

For a substance to be an appropriate food simulant for a particular food contact substance (FCS) under a particular condition of use, migration of the FCS into the prospective simulant under that condition should be the same as, or more than, its migration into the food it is intended to simulate. For milks and milk-like foods, 50% ethanol has recently been specified as a reasonable simulant for a wide range of organic migrants, despite it being too aggressive in some cases^38–40,77–80^. On the other hand, when the FCS is AgNPs, our data (Fig. 1) show that Ag migration into milk exceeded migration into 50% ethanol, particularly when the milk contained no fat. Therefore, the use of 50% ethanol may lead to underestimation of total Ag migration from AgNP-containing packaging materials. Additionally, our data (Fig. S3) also suggests that ethanolic simulants do not adequately capture the *form* of migrated Ag: Ag migrated into ethanolic solutions was almost completely ionic in nature, whereas Ag migrated into all milk contained Ag nanostructures of various sizes and compositions. This was, again, particularly the case in skim milk.

Before considering why ethanolic solutions underestimate Ag migration into milks, it is worth reviewing why simulants offer a reasonable reproduction of organic FCS in most cases. As noted in the introduction, food simulants generally work well for organic FCS because in a migration paradigm dominated by Fickian diffusion dynamics, the migrated mass in a finite system near equilibrium is largely determined by the food/polymer partition coefficient, which itself reflects the relative solubilities of the FCS in the food and in the polymer. For storage times that fall well short of the equilibrium point, the diffusion coefficient of the FCS in the polymer also plays an important role in determining migration; however, the contacted food rarely influences the diffusion coefficient of the FCS in the polymer except in cases where the food penetrates the polymer and causes swelling. As a practical matter, this is usually avoided when designing packaging. So, either near equilibrium or well before it, a food simulant usually only needs to replicate the solubility of the FCS in the food, providing a reasonable match to the food’s admittedly vaguely defined solubility properties (hydrophobicity, polarity, etc.). Importantly, typical models of FCS migration assume that the FCS undergoes no chemical changes either inside or outside the polymer that may substantially influence diffusion dynamics, which would include chemical interactions between the FCS and specific food components. If certain food components do exhibit strong interactions with the FCS, the usual food simulants, lacking such food components, may not be reliable to use in a migration assessment for the FCS. Accordingly, standard practices used to simplify migration experiments, like using food simulants, may need to be closely examined for use with newer classes of food contact materials; this is to ensure that these practices give a reliable facsimile of migration data that would be measured in real foods packaged within these novel materials.

The present study and our earlier work have demonstrated that polymer-bound AgNPs do not migrate like small organic molecules. Here, with milk, and previous studies with media containing sucrose^30^, TiO_2_^31^, and sulfides^32^, we have shown that chemical transformations between AgNPs, Ag^+^, and certain food substances influence the migration dynamics in ways that may not be accounted for when using simple solvents as food simulants. In the case of milk, migrated Ag^+^ readily changes form, engaging in a dynamic process that includes oxidation, reduction, particle seeding, and growth; these processes are sensitive to environmental conditions (light, oxygen content, ingredient profile). Analysis of specific interactions between Ag and key milk components, particularly whey, suggests that Ag⁺ ions exiting from the polymer are quickly adsorbed into protein structures naturally present in milk. In turn, these protein-Ag structural frameworks, and particularly subsequently formed m-AgNPs, deplete free Ag^+^ in the milk. This likely helps maintain a large concentration gradient at the polymer-milk interface, which maintains a large entropic driving force for diffusion of Ag⁺ out of the polymer. Additionally, Ag^+^ ions within the protein structures interact with reducing functionalities of amino acid residues, leading to disruptions in the secondary and tertiary protein structures^35,72,73^. Under aerobic environments, a large portion of protein-bound Ag readily converts to Ag₂O; on the other hand, some Ag^+^ remains in reduced form (Ag⁰), possibly due to poor penetration of oxygen into dense protein networks or mediation by available reducing reagents like lactose. In either case, bound Ag₂O or Ag⁰ form unstable nucleation sites that rapidly coalesce into larger Ag nanostructures as more Ag^+^ migrates across the polymer interface. The higher migration of Ag into whey and whey/lactose dispersions, compared to water and lactose solutions (Fig. S13 and Table S7), substantiates this dynamic process, suggesting that the Ag-protein interactions account for the higher Ag migration in milk compared to common milk simulants. The higher AgNP counts observed in the SP-ICP-MS time scans of protein dispersions compared to water and lactose solutions (Fig. S14) further support the efficient transformation of Ag⁺ ions to m-AgNPs in milk by proteins, even at trace Ag⁺ levels.

The protein content of the three milk types, skim, 2% fat, and whole milk, remains relatively consistent, with 8 g of protein per serving for both skim and 2% fat milk, and 7 g per serving for whole milk. However, the migration of Ag in whole milk was notably lower than in the other two milk types. This difference is likely attributed to the varying fat contents among the milks. Skim milk and 2% fat milk contain less than 0.5% and 2% fat, respectively, while whole milk contains 4% fat. Although in a strict sense, whole milk is more hydrophobic than skim milk, we questioned whether a few percent additional milkfat changes the overall hydrophobicity of milk to the degree that it could account for the substantial effect on Ag migration. On the other hand, both protein and fat exhibit a strong affinity for polymer surfaces^81–83^; in particular, we observed that the slightly higher fat content in whole milk created a much more substantial fat/protein layer adhering to the polymer surface, as judged by the difficulty rinsing the films prior to ICP-MS analysis. Therefore, we propose that strong adherence of milk fat and associated protein networks to the polymer surface acts as a barrier that slows the diffusion of Ag^+^ ions across the polymer-milk interface into the bulk milk. A modest increase in milkfat substantially increases the fat-and-protein barrier layer thickness on the polymer surface, accounting for the reduction of Ag migration we observed in whole milk versus 2% milk. Consequently, the Ag migration pattern observed in Fig. 1 reflects the combined influence of milk proteins and fats, with the water-soluble proteins in the bulk milk facilitating Ag^+^ migration out of the polymer and the fats and fat-soluble proteins impeding this process by forming a diffusion barrier at the polymer-milk interface.

Taken all together, this and our previous studies demonstrate that Ag^+^ sufficiently interacts with a variety of food components to affect Ag migration kinetics from AgNP-containing polymers in a substantial way. In milk, migrating Ag⁺ ions are rapidly bound into whey and casein protein structures. These Ag-protein interactions can then initiate the formation of both Ag^0^ and Ag_2_O nanostructures that deplete Ag^+^ concentrations in the milk and thus maximize the Ag^+^ concentration gradient across the polymer-milk interface. Milk fats, on the other hand, have strong adhesion to polymer surfaces, forming barrier layers that retard Ag migration out of the polymer. Neither of these effects are accounted for by ethanolic food simulants typically used for migration assessment with organic migrants from food contact polymers into milks or other dairy products. Importantly, this work also revealed key methodological steps needed to accurately assess Ag migration into foods, like milk, that contain large quantities of chloride ions and emulsified fats.

From a practical standpoint, our data show that the use of water or ethanolic solutions with low ethanolic content may provide a reasonable assessment of total Ag migration from AgNP-containing polymers into bovine milks under certain conditions. However, our data do *not* support the use of water or any ethanolic simulants to adequately determine the *form* of Ag migrated from these materials, as Ag migrated into milks exhibited a fraction of Ag nanostructures that were not observed in ethanolic or aqueous food simulants. From a broader standpoint, this study highlights how AgNPs and possibly other nanoparticle additives to polymers do not play by the same rules that organic migrants do. The fundamental physical and chemical principles that guide nanoparticle migration out of polymers appear to be quite different from those that direct the diffusion of small organic molecules. Accordingly, care should be taken to fully understand how migrated Ag or other redox-sensitive nanoparticle components may interact with specific food components before a food simulant is selected for migration testing. In several cases – notably sucrose, TiO_2_, and now milk proteins – we have observed that simple food simulants like water and ethanol may underestimate Ag migration.

## Methods

### Materials

Silver nitrate (AgNO_3_, 99.9999%), triethylene glycol (ReagentPlus®, 99%), dimethylamine (40% aqueous solution), poly(ethylene glycol) methyl ether thiol (average Mn 2,000, product number 729140), oleylamine (technical grade, 70%, product number 07805), whey protein (spray dried, product number W1500), casein (bovine milk, product number C7078), D-Lactose monohydrate (product number 61345), and LDPE pellets (product number 428043, density = 0.925 g mL-1at 25 °C, melt index = 25 g/10 min, MW ≈ 80 kDa) were purchased from Sigma-Aldrich. AgNP reference material (NanoXact Silver Nanospheres, product number: AGCN50-25M) was purchased from nanoComposix, Inc. Methanol (HPLC grade, 99.9%), ethanol (200 proof, anhydrous, Decon™ Labs), and toluene (HPLC grade) were purchased from Fisher Scientific. For all inductively coupled plasma optical emission spectrometry (ICP-OES) and mass spectrometry (ICP-MS) analyses, Optima™ grade nitric acid and hydrochloric acid (Fisher Scientific) were used. ICP silver (9980 ± 30 μg/mL in 4% HNO_3_, density = 1.032 g/mL @ 21.2 °C) standard was purchased from SCP Science (now AnalytiChem). Shelf-stable, ultra-high temperature (UHT) processed skim milk, 2% fat milk, whole milk, and whipping cream were purchased from Gossner Foods (Logan, UT). These milks and cream were purchased packaged in rectangular one-quart TetraPak containers primarily constructed of paperboard, plastic, and aluminum; the internal contact layer was polyethylene. Based on product labels, each 240 mL serving of skim milk and 240 mL serving of 2% fat milk contains 8 g protein, while a 240 mL serving of whole milk has 7 g of protein per serving. Skim milk has <0.5% fat while 2% fat milk, whole milk, and whipping cream contain 2%, 4%, and 33% fat, respectively.

All water for migration tests and chemical analysis was deionized to 18.2 MΩ⋅cm and dispensed from a Millipore-Sigma MilliQ Direct Q3 water purification system.

### Synthesis of AgNPs for AgNP/LDPE films

AgNPs incorporated into AgNP/LDPE films were manufactured through a two-step process involving synthesis in oleylamine (OA) and deep eutectic solvent (DES) and ligand exchange with poly(ethylene glycol) methyl ether thiol (PEG-SH-2000). The organic-phase synthesis of AgNPs utilizing OA as both a reducing agent and capping ligand offers enhanced control over the size distribution, stability, and yield of the metal NPs^41^. Consequently, this amine was employed for the high-throughput synthesis of OA-capped AgNPs. We choose to exchange OA with PEG-SH-2000 in this study to maintain consistency with our previous research. This approach allowed for the optimization of the well-controlled organic environment during synthesis, before modifying the NPs with the PEG thiol. For this, 160 mg (0.94 mmol) of AgNO_3_ was dissolved in 1.20 g (1.044 mL) of DES (1:1 Dimethylammonium nitrate:triethylene glycol) in a 10-mL reaction vial with a stir bar. 2 g (2.46 mL) of OA was then added to the vial, forming a homogeneous mixture. The mixture was stirred for 5 min at 25 °C and then heated at 150 °C in an oil bath for 25 min, resulting in a dark-brown colloid of AgNPs. The reaction was quenched by adding 10 mL of methanol after removing the vial from the oil bath. The resulting AgNPs were washed twice with 10 mL of methanol, using centrifugation (12,000 rpm, 5 min) to sediment the AgNPs. The residual solvent was removed using a gentle nitrogen gas flow. The dried AgNPs were then dispersed in 5 mL of toluene. The AgNPs had a mean diameter of 5.96 ± 1.15 nm, as determined by scanning tunneling electron microscopy (STEM). For ligand exchange, 0.5 mL of the AgNP in toluene was added to 0.2 g of PEG-SH-2000 pre-dissolved in 2 mL water. The mixture was gently vortexed and left undisturbed for 4 h to allow the ligand exchange to occur. Following this, the clear toluene layer was discarded, 2 mL of fresh toluene was added to the AgNP dispersion with a gentle vortex. After 1 h, the toluene layer was removed, leaving the AgNP dispersion in water in the vial, which was subsequently used for incorporation into LDPE films. UV–Visible spectra of AgNPs in both toluene and water are shown in Fig. S1A. The inset of Fig. S1A displays a photograph of AgNPs in toluene (left) and AgNPs undergoing the ligand exchange (right).

To evaluate the success of the ligand exchange process, we analyzed the AgNPs before and after the exchange using Fourier transform infrared (FTIR) spectroscopy. The FTIR spectra of PEG-SH-2000-AgNPs, both before and after ligand exchange, are shown in Fig. S2. The FTIR spectrum of AgNPs prior to ligand exchange exhibited characteristic bands, including the asymmetric C − H stretching and the in-plane bending vibration of cis-CH = CH- at 2920 and 727 cm⁻¹, respectively, in addition to the C-H stretch of the methylene groups at 2852 cm⁻¹, confirming the presence of OA on the AgNP surface^84^. After ligand exchange, these peaks disappeared, and new prominent bands attributed to the PEG groups, including the distinctive C–O–C stretch, appeared at 1098 cm⁻¹, suggesting a successful ligand exchange.

### Manufacture of AgNP/LDPE packaging

AgNP/LDPE films were prepared using a DSM Xplore micro-compounder with a 15 mL-barrel capacity equipped with a 65-mm cast film extrusion die. The micro-compounder and the die were heated to 150 °C and equilibrated with the screw speed set to 100 rpm with a maximum force tolerance of 10.75 N·m. Neat LDPE pellets (7 g) were added into the mixing chamber using a feed hopper and processed for 2 min. The screw speed was then set to 0 rpm, and 200 µL of aqueous AgNP solution, followed by a second batch of 7 g of neat LDPE pellets, was added into the mixing chamber. The mixture was then processed for another 6 min at the screw speed of 100 rpm at 10.75 N·m force tolerance. To produce the AgNP/LDPE film, the cast film extrusion die was first attached to the exit channel of the micro-compounder and the screw control was reduced to 0.95 N·m with a maximum speed setting of 100 rpm. The speed-controlled and torque-controlled rollers of the cast film extrusion die accessory were then set to values of 500 rpm and 28 N·m, respectively. The polymer melt exiting the film die was cooled with a nitrogen knife (11 L/min), pulled with tweezers, and guided onto the rollers for spooling. The films had a thickness of 50–52 µm and were rolled up and stored in the dark. A photograph of the AgNP/LDPE film is provided in Fig. S1B.

### Quantifying Ag in the AgNP/LDPE films

Approximately 150 mg of the AgNP/LDPE film was digested and analyzed using an iCAP PRO Series ICP-OES (ThermoFisher Scientific) using 2-stop, orange/white tubing (ID = 0.64 mm) with ASX-560 autosampler. Indium standard (5 ppm) was pumped into the sample introduction line with a 2-stop orange/yellow tubing (ID = 0.51 mm) using a Y-piece connector positioned after the peristaltic pump but before introduction into the nebulizer. The instrument was operated in iCAP PRO XP Duo configuration employing Aqueous, Axial, and instantaneous Full Range analysis (iFR) tune settings. Typical instrument parameters utilized for the experiments were as follows: RF power 1.15 W, nebulization gas pressure 0.13 bar, wash time 75 s, and uptake time 75 s. A calibration curve was produced using Ag standard solutions prepared by diluting Ag ion standards (10,000 μg/mL) from SCP Science (now AnalytiChem). A spectral line of 338.289 nm collected under axial mode was used to determine Ag concentration. Digestions were done in triplicate.

### Migration of Ag out of AgNP/LDPE film into milks and milk simulants

We followed a previously published protocol^42^ to assess the migration of Ag from AgNP/LDPE films into various types of milk. Extruded PC films were sectioned into 42 mm diameter circles using a hole punch and a hammer. The circles were rinsed three times in water to remove dust and polymer debris, blotted dry with a paper towel, and placed upright in 50 mL polypropylene tubes (Falcon centrifuge tubes, Thermo Fisher Scientific). Each tube was then filled with 25 mL of the test medium, which included either water, 10% aqueous ethanol, 50% aqueous ethanol, skim milk, 2% fat milk, or whole milk. The samples, prepared in quadruplicate, were stored at 20 °C for 10 days in a Thermo Max Q4000 orbital shaker set to 60 rpm. These conditions are recommended by the FDA in migration testing to simulate extended refrigerated storage conditions^29^. After 10 days, AgNP/LDPE circles from the milk samples were transferred to clean vials containing 10 mL ethanol and vortexed for 30 s to recover any milk residue adhering to the film surface. The films were removed, the ethanol rinse was evaporated, and the milk residues from the ethanol wash were combined with the milk prior to digestion. Each milk-plus-residue sample was then topped with 10 mL of concentrated nitric acid (HNO_3_) and the mixture was digested using a microwave digestion system (MARS 6, CEM, United States) equipped with a built-in method for food digestion. The digestion parameters included a 15 min ramp time, a 15 min hold time, a heating temperature of 210 °C, and a maximum pressure of 800 psi. Following digestion, 1 mL of concentrated hydrochloric acid (HCl) was added to the digestates to ensure complete dissolution of the Ag species. The final volume was then adjusted to 50 mL with deionized (DI) water before ICP-MS analysis. For the migration experiments involving aqueous dispersions of whey, lactose, and their mixtures, the films were rinsed with 10 mL (DI) water (instead of ethanol).

### Quantifying Ag migrated into milk and milk simulants

Migrated Ag was determined using an Agilent (Santa Clara, CA) 8800 triple quadrupole ICP-MS with a Micromist nebulizer and a Scott Double Pass spray chamber using single quadrupole MS mode. RF power and RF matching were set at 1550 W and 1.8 V, respectively. Carrier gas flow was 0.95 L/min. Sample uptake time was 30 s with 40 s stabilization time. Nebulizer pump speed was maintained at 0.5 rpm. Lenses were autotuned using 1 μg/L tuning solution from Agilent. For the internal standard, a 1000 μg/mL Rh internal standard mix from Agilent was diluted to 200 μg/L. A calibration curve was produced using Ag standard solutions prepared by diluting Ag ion standards (10,000 μg/mL) from SCP Science. A dilute acid stock solution was prepared by diluting 50 mL of concentrated HNO_3_ and 2 mL of concentrated HCl to a final volume of 1000 mL with deionized water, resulting in final concentrations of 5% (v/v) HNO_3_ and 0.2% (v/v) HCl. HCl provided a high concentration of Cl^-^ ions, facilitating the stabilization of Ag^+^ ions as AgCl_2_^-^ ions. The acid mixture was used for preparing standards, as well as for rinsing and as a blank solution during the analysis. The methodological limit of detection (LOD) and limit of quantitation (LOQ) for the ICP-MS analysis were calculated to be 7.66 ng/L and 8.27 ng/L, respectively. The Ag migration values for all the milks and milk simulants were measured above the LOD.

### Ag^+^ fate in milks and milk component dispersions

A 10 mL aliquot of each milk sample was transferred into a 20 mL glass vial (ThermoFisher Scientific) followed by the addition of 100 µL of AgNO_3_ stock solution (12.5 mg/mL). For the protein study, protein dispersions were prepared by dispersing 295 mg of casein powder or 63 mg of whey protein powder in 10 mL of DI water. The suspensions were vortexed, resulting to an almost clear solution for whey, while casein remained only partially dissolved. To assess the effect of lactose on the formation and composition of Ag nanostructures, additional protein dispersions were prepared that included 500 mg of lactose powder. Then, 100 µL of the AgNO_3_ stock solution was added to each protein mixture. The vials were placed in a Thermo Max Q4000 orbital shaker set at 60 rpm and 20 °C. Samples were incubated for 10 days, during which they were monitored for visual color changes. These color changes were periodically captured using a digital camera. To further characterize the formation of Ag nanostructures in the protein mixtures, the particles were centrifuged at 15000 rpm for 5 min, re-dispersed in fresh DI water, and analyzed by X-ray diffraction (XRD) and UV-Vis spectroscopy.

The formation of Ag nanostructures in aqueous whey dispersions containing trace concentrations of Ag⁺ ions was monitored using ICP-MS in single-particle mode (SP-ICP-MS). A 25 mL whey dispersion (6.3 g/L) was spiked with an AgNO₃ stock solution (100 mg/L) to achieve final Ag⁺ concentrations of 100 ng/L, 500 ng/L, or 1000 ng/L. A negative control consisting of 100 ng/L Ag^+^ ions in water without whey was included. The mixtures were vortexed and incubated at 20 °C with gentle agitation on an orbital shaker (60 rpm) for several days. At specific time intervals, 1 mL aliquots were withdrawn, mixed with 9 mL of DI water, vortexed, and sonicated for 5 min. These samples were then analyzed by SP-ICP-MS using an Agilent 8800 triple quadrupole ICP-MS (Santa Clara, CA) in time-resolved analysis (TRA) mode, with an integration time of 3 ms. The samples were introduced to the ICP-MS via an ASX-500 series autosampler, with a flow rate to the nebulizer of 0.346 mL/min. AgNP reference material (50 nm diameter, 10 ng/L) was diluted from commercial AgNPs (50 nm, 0.02 mg/mL) purchased from nanoComposix, Inc. Sample uptake time was 120 s with 0.1 rpm nebulizer pump speed and 30 s stabilization. Data analysis was performed using ICP-MS MassHunter software. Particle baseline and detection threshold were automatically determined in the MassHunter software.

### Adsorption of Ag^+^ ions onto whey protein

Whey dispersion (25 mL, either 6.3 g/L or 0.2 g/L) was mixed with an equal volume of AgNO₃ solution (200 mg/L), resulting in a final Ag⁺ concentration of 100 ng/L. The mixture was gently mixed and incubated at 20 °C with agitation at 60 rpm on a temperature-controlled orbital shaker. 1.5 mL aliquots were collected at specific time intervals and centrifuged at 5000 rpm for 5 min. 1 mL of the supernatant was diluted to 10 mL with 5% HNO₃ for Ag analysis using ICP-MS in standard mode.

### Dynamic light scattering (DLS) analysis of whey dispersions

Hydrodynamic diameters were measured on a Malvern Particle Size Analyzer. Whey powder was dispersed in either purified water or phosphate buffer solution (pH 7.0, as received) at a concentration of 0.025 wt %, followed by vortexing for 1 min to ensure homogeneity. To investigate the interaction between whey proteins and Ag^+^ ions, 100 µL of AgNO₃ solution (0.49 mg/mL) was added to 10 mL of the whey dispersion and gently mixed. Measurements were performed in quartz cuvettes. The solvent viscosity, refractive index, and other relevant optical parameters were set to the default values provided by the instrument software (Malvern Zetasizer Software, version 2012). Each sample was equilibrated for 120 s prior to analysis, followed by 15 consecutive measurements without disturbing the dispersion between runs.

### UV–Visible spectroscopy

UV–Visible spectra of the AgNPs formed in various milks and proteins were acquired on a Agilent Cary 3500 UV-Visible Spectrophotometer. After a specified incubation period, 0.5 mL of each storage solution was diluted to 3.5 mL (7-fold dilution) and transferred to a 1-cm path length quartz cuvette (Starna). The samples were immediately scanned with a 1 nm slit width at 480 nm/min over a range of 300-800 nm using neat milk as the reference. For the chemically synthesized AgNPs, a 5 µL aliquot of the AgNP dispersion was diluted to 3 mL in a 1-cm path length quartz cuvette and scanned with a 1 nm slit width at 480 nm/min over a range of 300–700 nm, with neat toluene as the blank.

### Transmission electron microscopy (TEM)

TEM images of the AgNPs formed in various milk and protein solutions were recorded with a JEOL 3200FS TEM with a Gatan K2 detector at an acceleration voltage of 300 kV at the BioCryo Facility of NUANCE at Northwestern University (Evanston, IL). TEM samples were prepared similarly to those for UV-Visible analysis, with the additional centrifugation and re-dispersion steps to remove the majority of the unbound milk matrix from the AgNP surfaces.

### Fourier transform infrared spectroscopy (FTIR)

FTIR spectra were recorded using a PerkinElmer Frontier FTIR/NIR Spectrophotometer, with data acquisition controlled by Spectrum IR software. The measurements were conducted in transmission mode, utilizing the Mid-Infrared (MIR) range. The spectral scan range was set from 4000 to 600 cm⁻¹, with a total of 32 scans per sample to enhance signal quality. A diamond crystal ATR accessory was used as the optical element for the measurements. After background correction, samples of OA, PEG-SH, and ligand-capped AgNPs were directly applied to the diamond crystal surface and dried under a fan to ensure proper adhesion and solvent removal before the spectra were recorded.

### X-ray Diffraction (XRD)

XRD analysis was performed on a Brüker D2 Phaser with a copper anode (0.1542 nm wavelength) as the X-ray source. The samples prepared for TEM imaging were also used for the diffraction studies. A 50 µL droplet of concentrated AgNP dispersion was drop-cast onto a polished silicon wafer, allowed to air-dry, and then placed in the sample holder. To minimize scattering interference, a 3.0 mm air scattering screen, 0.6 mm divergence slit, and 1 mm detector slit were employed. The samples were rotated at 15 rpm during measurement, with data collected over a 2θ angular range of 20–85 degrees and a 20 s integration time. Baselines and scattering contributions from Cu Kα were subtracted from the raw data using Brüker D2 Phaser DIFFRAC.EVA software with automated functions.

### Raman analysis

Raman analysis of the AgNPs was performed using a Horiba XploRA PLUS confocal Raman microscope equipped with a 785 nm excitation laser and ×100 objective lens. Raman spectra was acquired with a 1200 gr/mm grating and 500 s total acquisition time. m-AgNPs formed in a whey protein solution stored in the presence of 0.73 mM AgNO_3_ at 20 °C for 10 days were centrifuged, washed three times, and redispersed in fresh deionized water. A 50 µL aliquot of the dispersion was then drop-cast onto a smooth steel plate and air-dried for Raman analysis. The collected spectra were processed to remove baseline using Labspec 6 software.

### Additional information about development of analytical methods for Ag in milk

Method development focused on optimizing the conditions for the migration tests as well as maximizing recovery of Ag in milks after the migration tests were completed. Migration tests initially used sachets (small enclosed packages containing simulant) to avoid edge effects commonly observed when immersing punched sections of the test material in simulants. However, we encountered significant challenges with sachets due to the strong adherence of milk to the interior surfaces of the sachets, which complicated sample recovery. Consequently, we shifted to using 42 mm-diameter punched circles in immersion tests with milk. Recovery of milk adhered to the circles after they were extracted from milk was accomplished by vortexing the extracted circles with clean media in a separate tube. Sample storage tubes were also rinsed with the same media. All the rinses were then retained for digestion and analysis along with the milk. Initially, we used water as the rinse solution to recover residual milk adhering to the film circles and storage tube surfaces. However, cloudy residue, presumably consisting of fat and protein, remained on the film and tube surfaces after water rinses, and we observed inconsistencies in the Ag migration between experiments. We determined that water was not an effective rinse solution for milk, as it does not dissolve the milk residue sufficiently. To address this issue, we tested a range of solvents, including ethanol, acetone, and isopropanol. Ethanol proved to be the most suitable rinse solution in terms of consistency of the migration data and ability to remove milk residue from the plastic surface while exhibiting minimal penetration into the LDPE matrix.

Even with the adjustments to the protocol for processing milk samples, migrated levels of Ag still exhibited unacceptable inter-sample variability. Upon closer examination of the composition of bovine milk, we discovered that milk contains approximately 0.8 to 1.2 g/L Cl⁻ ions^50^. We hypothesized that these ions react with Ag⁺ ions, forming stable silver chloride (AgCl) precipitate, which can interfere with the determination of dilute Ag concentrations by ICP-MS. To mitigate this, we added 1 mL of concentrated hydrochloric acid (HCl) to the digestates prior to dilution and introduction into the ICP-MS nebulizer. This fortification with Cl⁻ ions helped maintain the necessary Cl^–^ concentration to promote soluble AgCl_x_^(x-1)−^ complexes formation^47^ and prevent AgCl precipitation. These refinements led to improved consistency in the migration results.

Finally, while ethanol rinses did a satisfactory job of removing milk residues from the film and tube surfaces, we also discovered that migrated Ag⁺ ions were still adhering to the surfaces of the migration cell (50-mL polypropylene tubes). The most consistent results were obtained when sample tubes were thoroughly rinsed with a concentrated nitric acid (HNO₃) solution to recover any Ag species that may have adsorbed on the migration cell walls. We also found improved results when entire migration solutions, rather than aliquots, were used to determine migration due to inhomogeneous distribution of Ag in the migration cell (free in solution, adhered to tube walls, etc.). While we have since adopted the strategy of avoiding aliquots for all analyses of Ag migration due to how easily Ag adsorbs to plastic surfaces, this seems to be particularly important when analyzing milk, as Ag tends to bind preferentially to plastic surfaces in the presence of milkfat.

## Supplementary information

Supplementary Information

## Data Availability

Experimental (ICP-MS and SP-ICP-MS) migration data are provided in table format in the Supporting Information section. Data used to generate X-ray diffractograms and Raman/UV–Vis spectra are available upon request to the corresponding author.
