# Supplementary material for: Milk proteins and fat influence Ag migration from model dairy packaging containing silver nanoparticles
Source: NPJ Sci Food. 2026 Jan 13;10:36. doi: 10.1038/s41538-025-00684-5 (PMC12881427; doi:10.1038/s41538-025-00684-5)
Supplement: Supplementary file 1 — Supplementary Information [file 41538_2025_684_MOESM1_ESM.pdf]

Milk proteins and fat influence Ag migration from model dairy packaging containing silver nanoparticles

Laxmi Adhikari[1], Srushti B. Pansare[2], Rakesh R. Mudireddy[2], Monisha Srinivasan[2], Timothy V. Duncan[1*]

[1]Human Foods Program, US Food and Drug Administration, Bedford Park, IL 60501

[2]Department of Food Science and Nutrition, Illinois Institute of Technology, Bedford Park IL, 60501

*Email: timothy.duncan@fda.hhs.gov

Supporting Tables

Table S1. pH values of milk samples with and without 650 ppt Ag^+ ions over a 10-day period, stored under refrigeration (4 °C) and at room temperature (20 °C).[a]

	2% fat milk		2% fat milk with 650 ppt Ag^+	
	5 °C	20 °C	5 °C	20 °C
Day 0	6.40 ± 0.01	6.40 ± 0.01	6.40 ± 0.01	6.40 ± 0.01
Day 7	5.53 ± 0.01	6.47± 0.01	6.54 ± 0.01	6.52 ± 0.01
Day 10	6.56 ± 0.01	6.51± 0.02	6.56 ± 0.01	6.53 ± 0.01

[a]Each sample consistent of 25 mL of 2% fat milk (from a freshly opened container) in 50 mL polypropylene centrifuge tubes (Falcon, Fisher Scientific). All samples were prepared in triplicate, and independent sample sets were used for each time point.

Table S2. Ag migration from AgNP/LDPE nanocomposite sections into water, 10% ethanol, 50% ethanol, and various bovine milks after 10 days of storage at 20 °C. These data were used to construct Figure 1.

**Simulant/milk**	**Ag release (ng/cm^2)**
Water	2.025 ± 0.146
10% EtOH	1.988 ± 0.168
50% EtOH	1.288 ± 0.053
Skim milk	2.223 ± 0.099
2% fat milk	2.181 ± 0.032
Whole milk	1.780 ± 0.068

Table S3. Free Ag^+ ions concentration in solution after mixing $AgNO_3$ solution into 25 mL whey dispersion, resulting in a final Ag^+ concentration of 100 ng/L. These data were used to construct Figure S9. One data point at 12 minutes is missing due to human error.

Time (min)	2000 mg/L of whey (ng/L)	200 mg/L of whey(ng/L)
0	100.00	107.44
2	21.01	62.66
7	19.59	62.07
12	17.77	
20	16.77	60.59
60	15.82	60.28
120	16.92	60.29

Table S4. SP-ICP-MS analysis of AgNPs formed in whey dispersions (6.3 g/L) spiked with different concentrations of Ag^+ ions: particle number concentration, particle mass concentration, background equivalent diameter (BED), and mean particle diameter are shown after storage at 20 °C. These data were used to construct Figure 6.

Incubation time	Particle number (×10^6/L)			
	**Control-100 ppt**	**Whey-100 ppt**	**Whey-500 ppt**	**Whey-1000 ppt**
48 h	2 ± 1	33 ± 4	58 ± 2	77 ± 7
72 h	1 ± 0	35 ± 2	69 ± 3	100 ± 6
144 h	1 ± 0	39 ± 5	63 ± 1	67 ± 2
	**Mass concentration (ng/L)**			
	**Control-100 ppt**	**Whey-100 ppt**	**Whey-500 ppt**	**Whey-1000 ppt**
48 h	0.31 ± 0.17	5.77 ± 1.07	14.74 ± 1.87	18.84 ± 1.44
72 h	0.20 ± 0.03	5.95 ± 0.77	16.61 ± 0.82	19.49 ± 0.13
144 h	0.11 ± 0.03	5.76 ± 0.77	12.53 ± 1.52	10.79 ± 0.56
	**BED (nm)**			
	**Control-100 ppt**	**Whey-100 ppt**	**Whey-500 ppt**	**Whey-1000 ppt**
48 h	8.0 ± 0.1	12.2 ± 0.8	20.0 ± 0.1	25.1 ± 0.4
72 h	7.8 ± 0.1	12.3 ± 0.5	20.1 ± 0.1	24.7 ± 0.1
144 h	7.7 ± 0.1	12.2 ± 0.7	18.5 ± 0.6	25.0 ± 0.2
	**Mean size (nm)**			
	**Control-100 ppt**	**Whey-100 ppt**	**Whey-500 ppt**	**Whey-1000 ppt**
48 h	28.2 ± 8.8	27.1 ± 0.4	31.2 ± 0.7	31.0 ± 0.8
72 h	32.0 ± 7.4	26.1 ± 0.7	30.3 ± 0.4	29.6 ± 0.4
144 h	27.7 ± 4.1	25.2 ± 1.4	28.7 ± 1.1	27.9 ± 0.2

Table S5. Dynamic Light Scattering (DLS) measurements of aqueous dispersions of commercial whey protein powder (0.025 wt %) in purified water over time following the introduction of Ag^+ ions. The table reports the Z-average hydrodynamic diameter (intensity-weighted mean size, in nm) and the area intensities (%) and associated mean particle diameters of the largest two peaks in the DLS spectrum (Pk 1 and Pk 2). Measurements were taken at different incubation times (0–48 h) to monitor whey protein aggregate evolution in the presence of Ag. See Figure S10 representative plots of the DLS data at the 0, 24, and 48 hr time points.

Time (h)	Z-average diameter (nm)	Pk 1 Area Int (%)	Pk 1 Mean Int (d.nm)	Pk 2 Area Int (%)	Pk 2 Mean Int (d.nm)
0	359	8	28	92	227
1	348	10	35	90	236
2	322	11	36	89	272
3	320	11	35	90	220
4	264	10	33	91	241
5	312	16	40	84	231
6	277	13	41	87	234
7	275	18	39	82	249
8	271	16	32	84	206
24	93	42	28	51	120
48	36	87	52	11	9

Table S6. Dynamic Light Scattering (DLS) measurements of aqueous dispersions of commercial whey protein powder (0.025 wt %) in pH 7.0 buffer solution over time following the introduction of Ag^+ ions. The table reports the Z-average hydrodynamic diameter (intensity-weighted mean size, in nm) and the area intensities (%) and associated mean particle diameters of the largest two peaks in the DLS spectrum (Pk 1 and Pk 2). Measurements were taken at different incubation times (0–48 h) to monitor whey protein aggregate evolution in the presence of Ag. See Figure S11 shows representative plots of the DLS data at the 0, 5, and 7 hr time points.

Time (h)	Z-average diameter (nm)	Pk 1 Area Int (%)	Pk 1 Mean Int (d.nm)	Pk 2 Area Int (%)	Pk 2 Mean Int (d.nm)
0	240	14	40	86	207
1	232	21	48	79	219
2	211	19	45	81	210
3	184	27	53	73	233
4	172	33	50	67	193
5	117	59	65	41	238
6	105	55	60	45	157
7	96	100	87	0	0
8	82	94	97	4	18
24	87	100	79	0	0
48	81	100	91	0	0

Table S7. Ag migration from AgNP/LDPE film sections into water, 5% lactose, whey (6.5 g/L), and lactose/whey (5%/6.5 g/L) dispersions after 10 days of storage at 20 °C. These data were used to construct Figure S11.

**Simulant/protein**	**Ag release (ng/cm^2)**
Water	1.060 ± 0.070
Lactose	1.357 ± 0.046
Whey	2.228 ± 0.083
Whey + lactose	2.136 ± 0.017

Figures

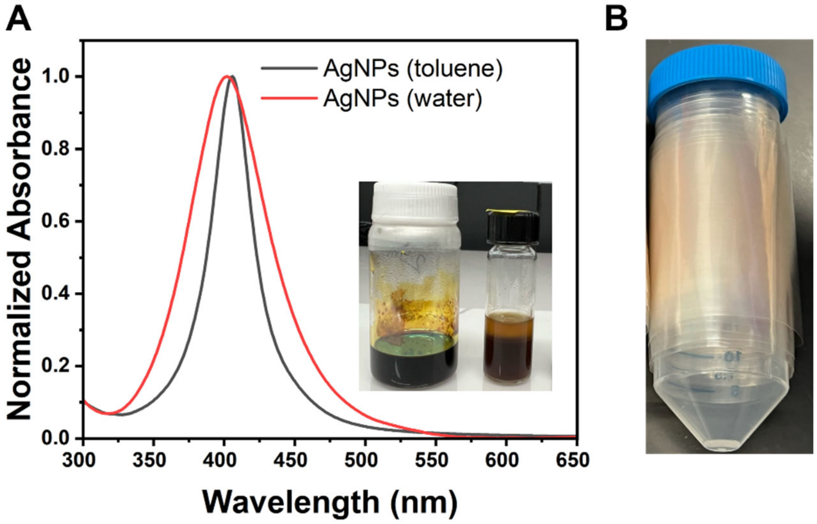

Figure S1. Photographs and characterization data for OA- and PEG-SH-2000-capped AgNPs and AgNP/LDPE film. (A) Normalized UV–Visible spectra of AgNPs produced by reducing $AgNO_3$ with OA. The OA-capped AgNPs dispersed in toluene (black line) were later ligand exchanged with PEG-SH-2000 and dispersed in water (red line). The inset in **panel A** shows a photograph of AgNPs in toluene (left) and AgNPs undergoing the ligand exchange (right). (B) Photograph of PEG-SH-2000-AgNP/LDPE film used in this study.

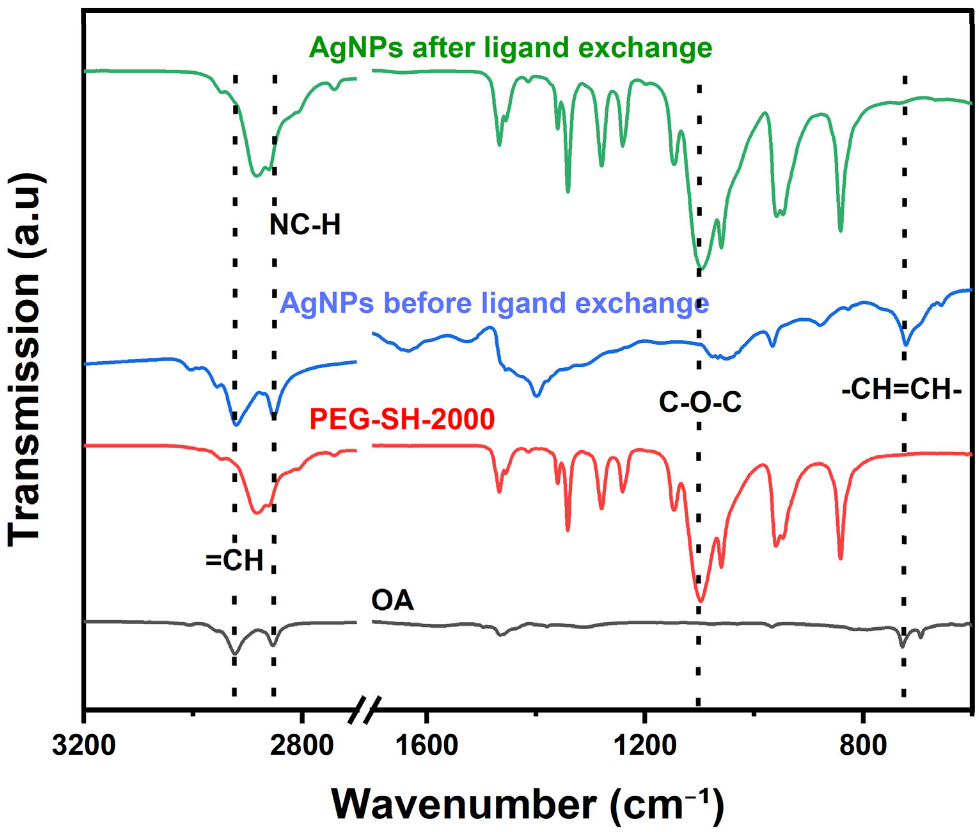

Figure S2. FTIR spectra of OA (black line), PEG-SH-2000 (red line), and AgNPs before (blue line), and after (green line) ligand exchange with PEG-SH-2000.

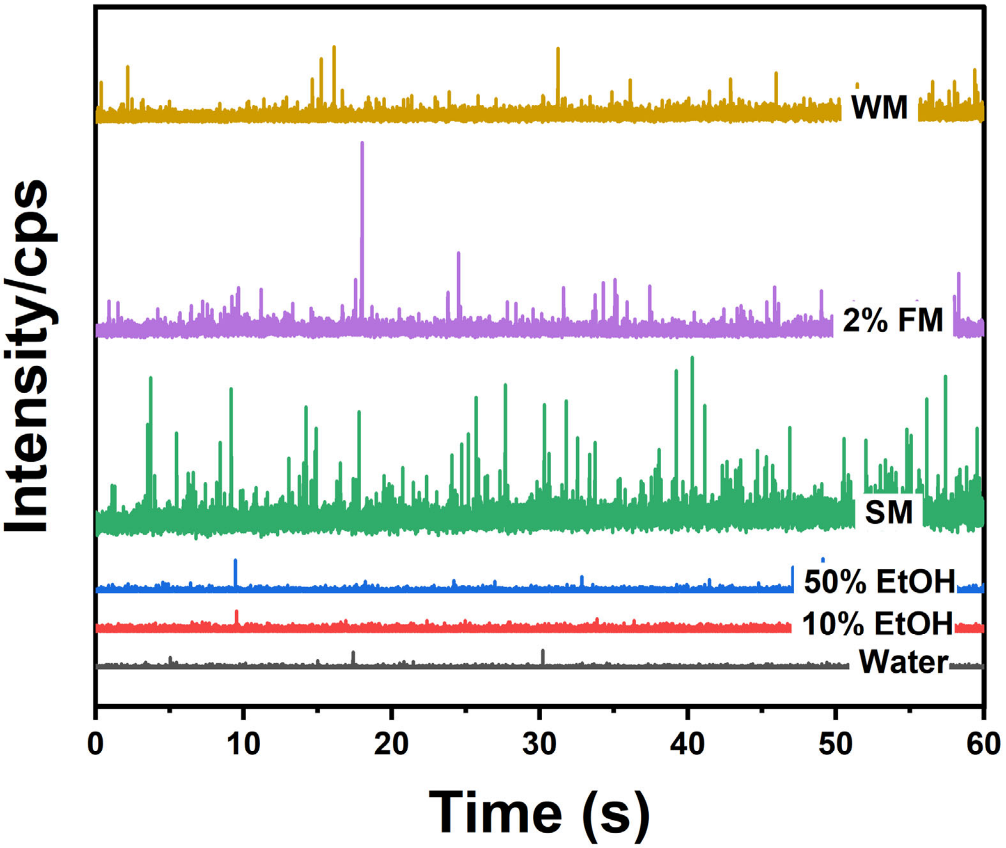

Figure S3. Representative SP-ICP-MS time scan of ^{107}Ag for migration samples, including water (black), 10% aqueous ethanol (red), 50% aqueous ethanol (blue), skim milk (green), 2% fat milk (purple) and whole milk (yellow), stored with AgNP/LDPE film section for 10 days at 20 ºC. A 100 µL aliquot of each migration solution was diluted to 50 mL for analysis. The time scan demonstrates the presence of a small number of NPs in water and ethanol solutions, while significantly higher particle counts were observed in the milk samples.

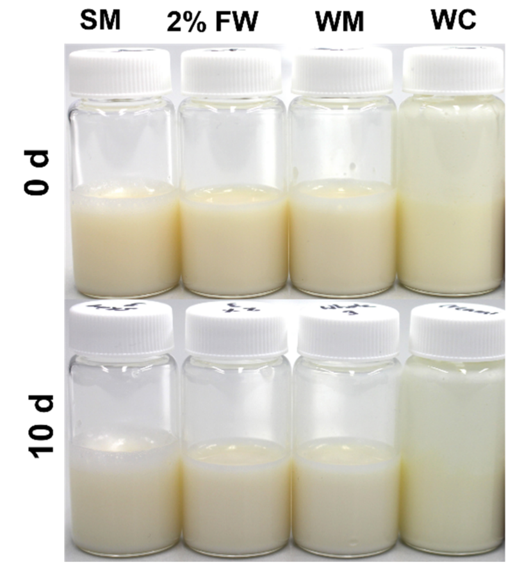

Figure S4. Photographs of milk samples taken on Day 0 and Day 10 after following a $AgNO_3$ spike and storage in the dark. Each milk type (SM: skim milk, 2% FM: 2% fat milk, WM: whole milk, WC: whipping cream) was spiked with 100 μL of $AgNO_3$ stock solution (12.5 mg/mL) and gently vortexed for 5 seconds. The vials were placed in a Thermo Max Q4000 orbital shaker set at 60 rpm and 20 °C, inside a carboy to prevent light exposure. No visible color change was observed over the 10-day period, indicating that Ag^+ reduction did not occur under dark conditions.

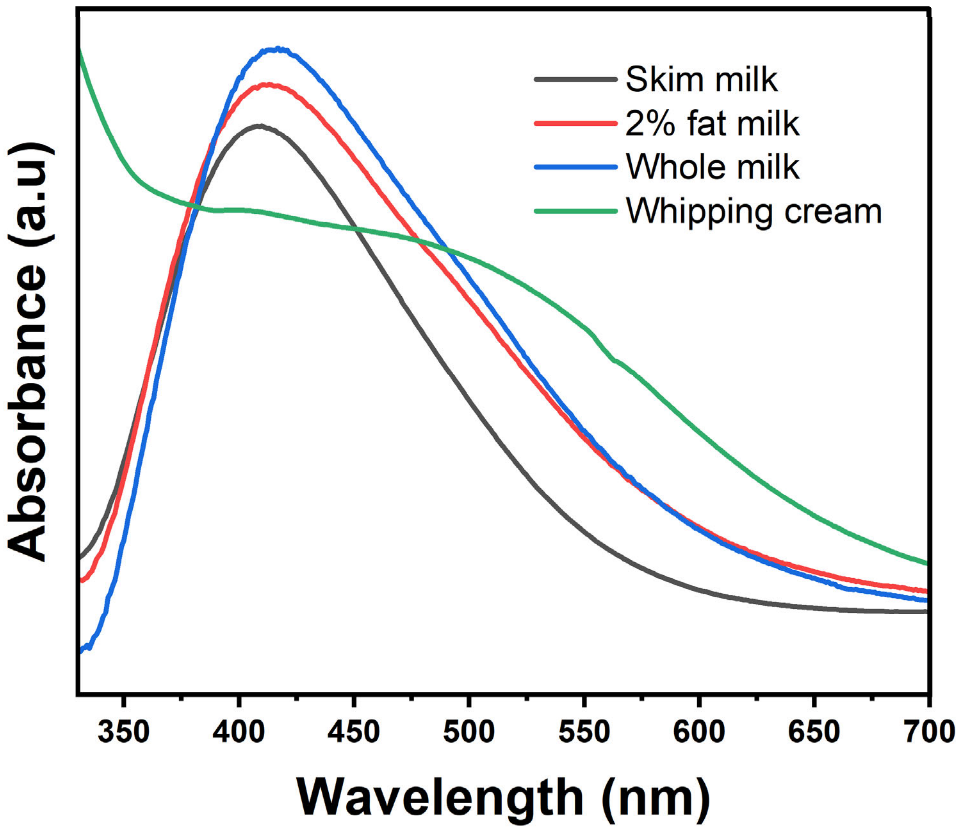

Figure S5. UV–Visible spectra of AgNPs isolated from milks 3 days after spiking with $AgNO_3$. The broad surface plasmon resonance (SPR) bands at the UV and visible region suggest the presence of m-AgNPs in the milk extracts. The maximum absorbance wavelengths (λ_{max}) were observed at 410 nm, 413 nm, and 417 nm for m-AgNPs in skim, 2% fat, and whole milk, respectively, suggesting a trend of larger m-AgNP formation with higher fat content.[1] The SPR signatures of m-AgNPs obtained in whipping cream was too broad for λ_{max} determination but still provide evidence of m-AgNPs formation.

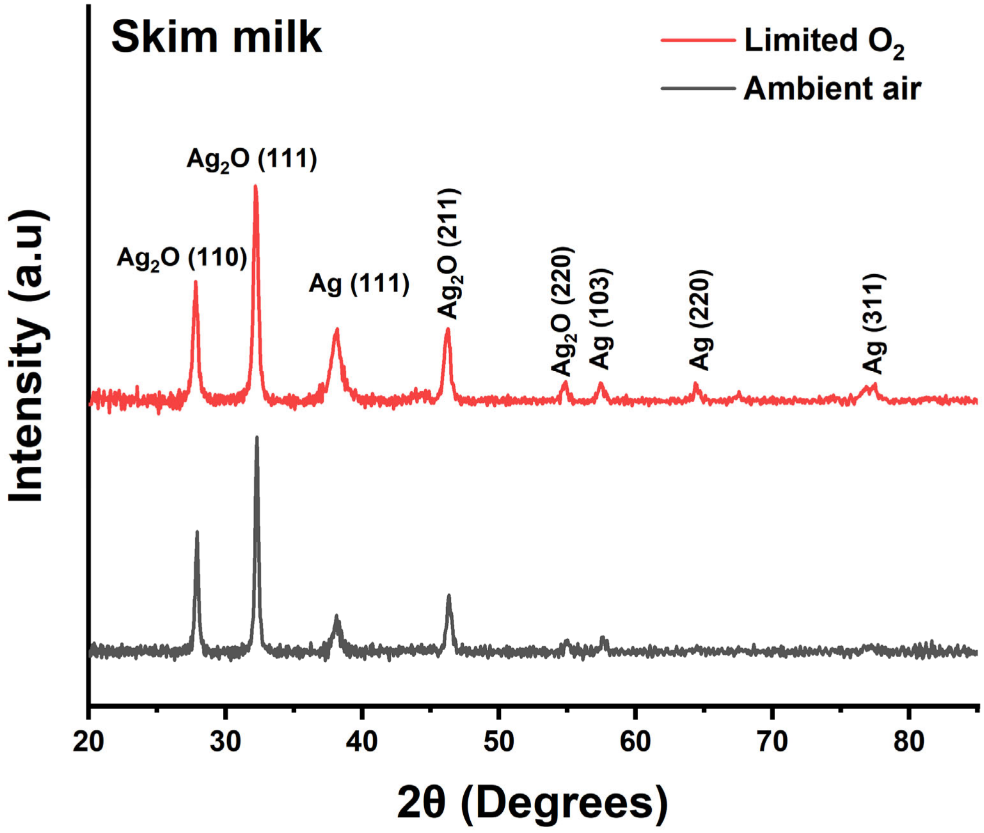

Figure S6. X-ray diffractograms of m-AgNPs isolated from skim milk 10 days after spiking with $AgNO_3$. Skim milk was either used directly from the bottle (black line) or purged with a gentle flow of N_2 for 30 min (red line) prior to Ag^+ ion spiking and incubation. m-AgNPs isolated after exposure to limited oxygen (N_2 purged, red line) exhibited enhancement of peaks associated with metallic Ag^0 NPs compared to m-AgNPs formed under ambient air (black line).

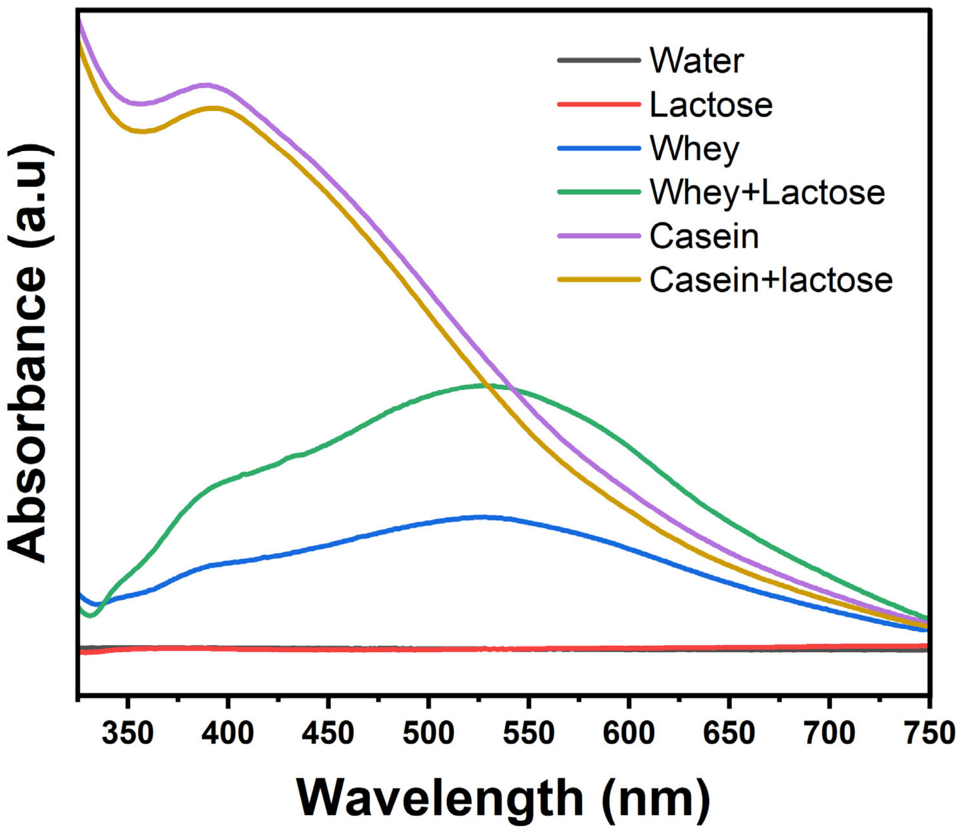

Figure S7. UV–Visible spectra of m-AgNPs isolated from lactose and protein (casein and whey) dispersions, 24 h after spiking with $AgNO_3$. The broad SPR bands at the UV and visible region suggest the presence of AgNPs in the protein extracts. In contrast, no SPR band was observed for the lactose isolate, confirming minimal formation of m-AgNPs in the lactose solution.

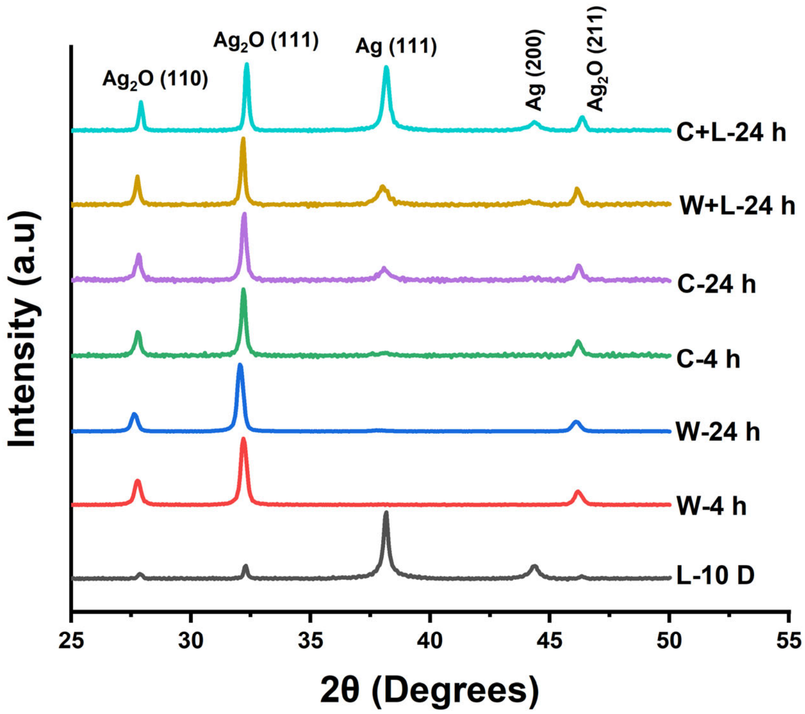

F**igure S8.** X-ray diffractograms of m-AgNPs isolated from lactose and protein solutions spiked with $AgNO_3$ and incubated for varying periods at 20 °C. The diffractograms reveal that both milk proteins, casein and whey, facilitated the formation of Ag_2O nanostructures, while lactose promoted the formation of predominantly metallic Ag^0 NPs. In protein-lactose mixtures, lactose played a crucial role in shifting the composition towards more Ag^0 NPs, potentially favoring Ag^+ reduction.

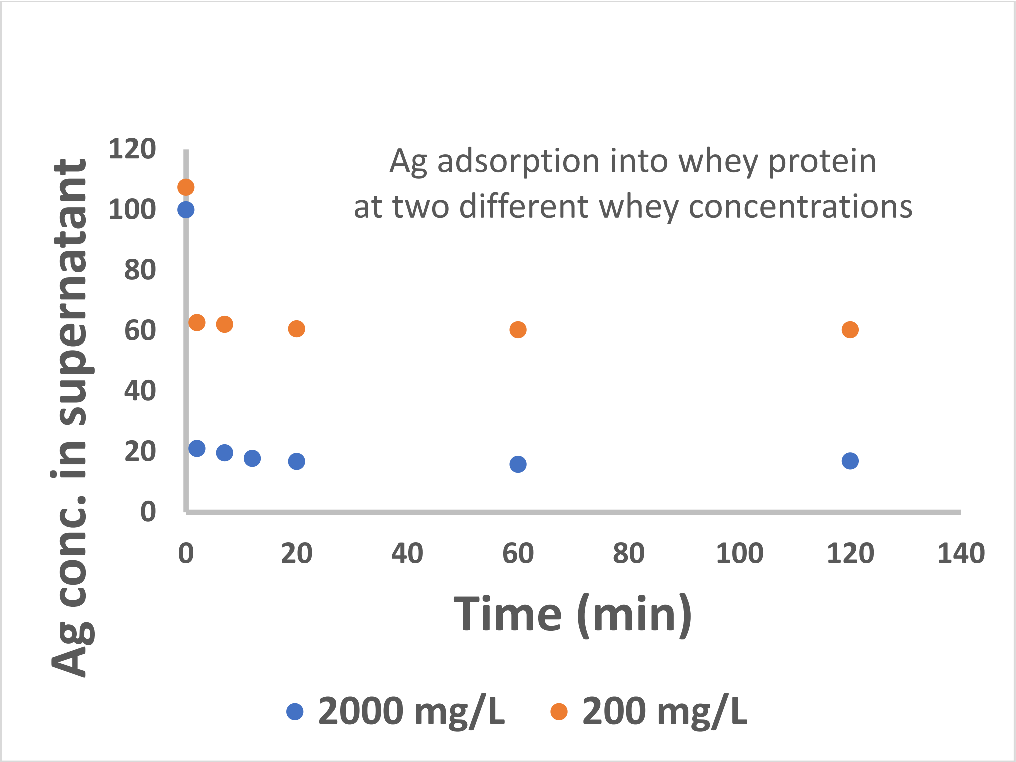

Figure S9. Ag^+ ions sorption onto whey protein solutions at two different concentrations. A 25 mL whey solution (either 2000 mg/L or 200 mg/L) was mixed with an equal volume of $AgNO_3$ solution (200 mg/L), resulting in a final Ag^+ concentration of 100 ng/L. The initial free Ag^+ concentration (the amount of Ag^+ left in the supernatant after centrifugation to remove the protein-Ag^+ network) of 100 ng/L decreased to approximately 63 ng/L and 21 ng/L for the 200 mg/L and 2000 mg/L whey solutions, respectively, within 2 minutes. After this initial decrease, the concentrations of free Ag^+ remained relatively stable, indicating fast binding kinetics and strong Ag^+ absorption onto the protein structure.

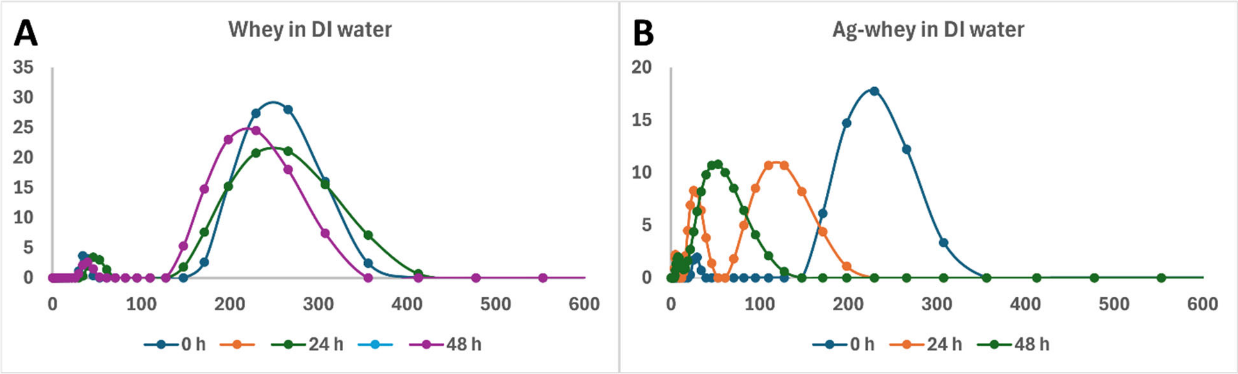

Figure S10. Representative plots of DLS data illustrating the evolution of whey particle size distribution over time in dispersions of commercial whey protein powder (0.025 wt %) in purified water (A) without and (B) with the addition of Ag^+ ions.

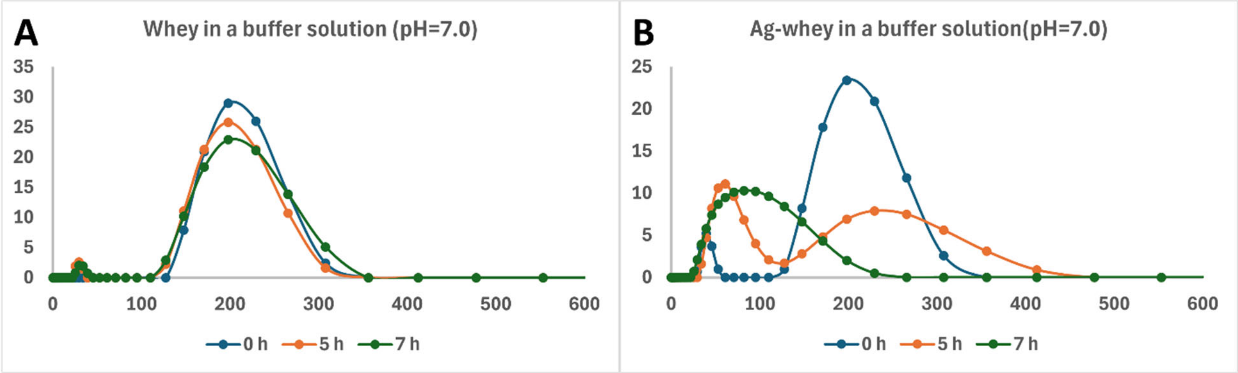

Figure S11. DLS graphs illustrating the evolution of whey particle size distribution over time in dispersions of commercial whey protein powder (0.025 wt %) in pH 7.0 phosphate buffer (A) without and (B) with the addition of Ag^+ ions.

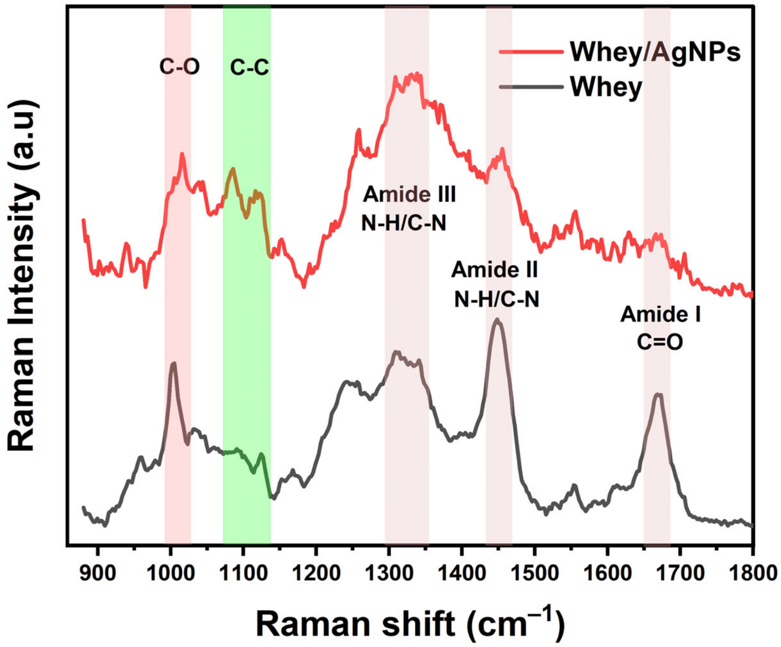

Figure S12. Raman spectra of m-AgNPs isolated from a whey protein dispersion stored in the presence of 0.73 mM $AgNO_3$ at 20 ºC for 10 days.

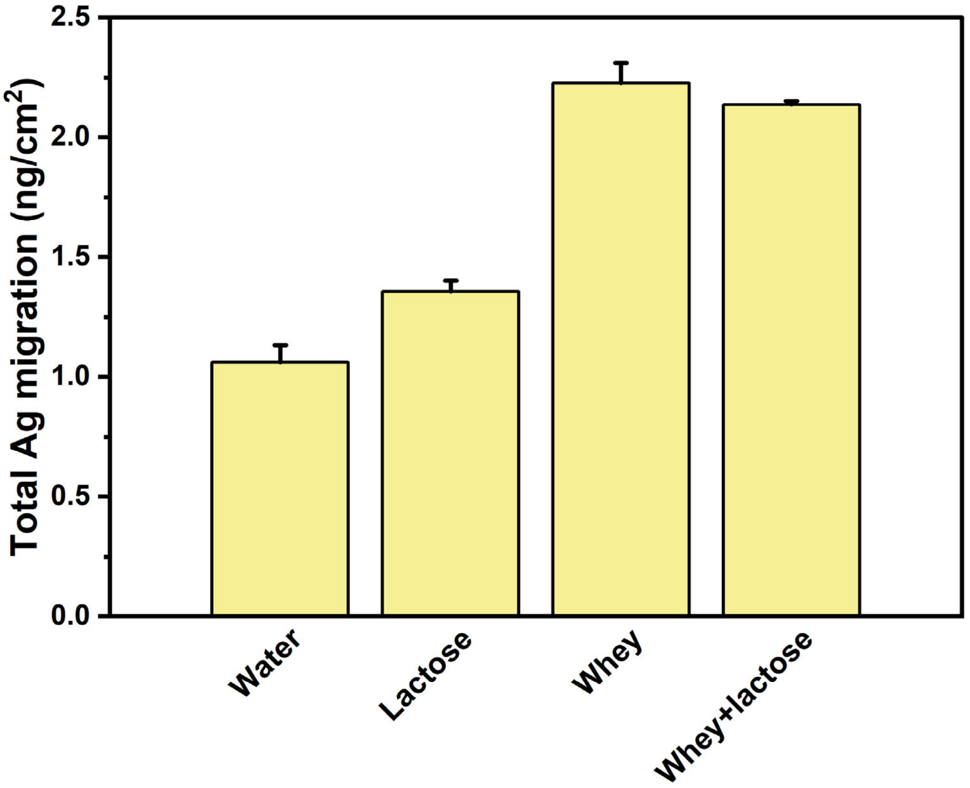

Figure S13. Ag migration from AgNP/LDPE film sections into water, 5% lactose, whey (6.5 g/L), and lactose/whey dispersions after 10 days of storage at 20 °C. The PNC samples were sectioned into 42 mm diameter circles, and 25 mL of each simulant was used for the migration study. All data points represent the averages of four independent samples, with error bars indicating standard deviations.

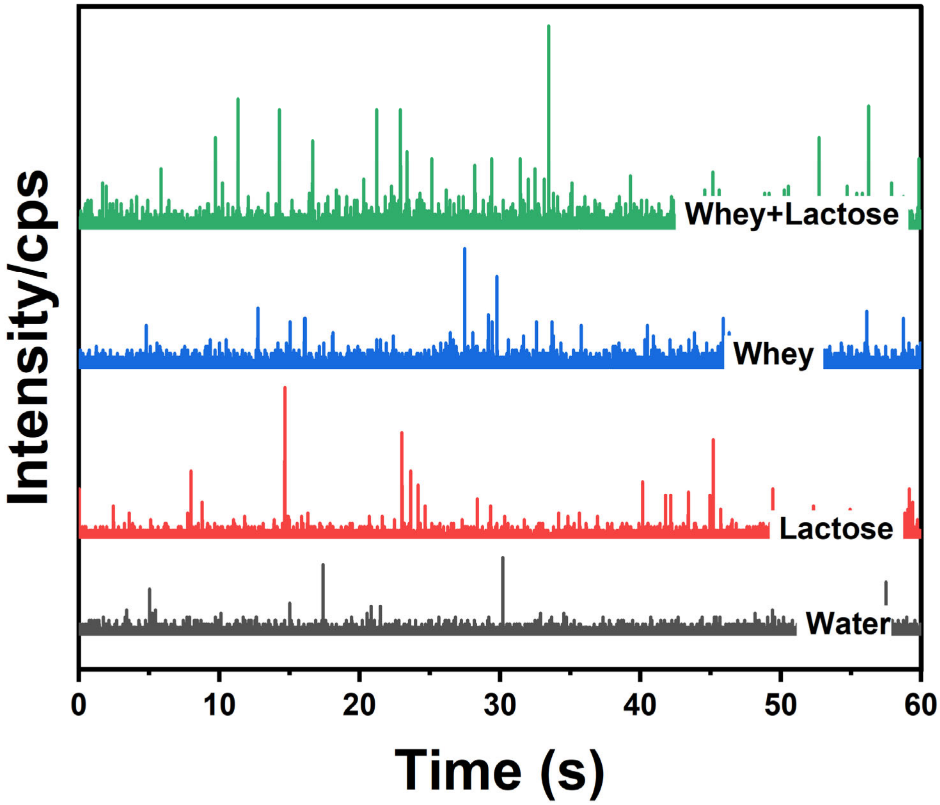

Figure S14. Representative SP-ICP-MS time scan of ^{107}Ag for migration samples, including water (black), 5% lactose (red), whey (blue) and whey/lactose dispersions (green), stored with AgNP/LDPE film section for 10 days at 20 ºC. A 100 µL aliquot of each migration solution was diluted to 50 mL for analysis. The time scan demonstrates the presence of a small number of NPs in water, while higher particle counts were observed in lactose solution and protein dispersions.

References

(1) Kravets, V.; Almemar, Z.; Jiang, K.; Culhane, K.; Machado, R.; Hagen, G.; Kotko, A.; Dmytruk, I.; Spendier, K.; Pinchuk, A. Imaging of Biological Cells Using Luminescent Silver Nanoparticles. *Nanoscale Res. Lett.* **2016**, *11* (1), 30. DOI: 10.1186/s11671-016-1243-x.
